# Malaria Parasite Stress Tolerance Is Regulated by DNMT2-Mediated tRNA Cytosine Methylation

**DOI:** 10.1128/mBio.02558-21

**Published:** 2021-11-02

**Authors:** Elie Hammam, Ameya Sinha, Sebastian Baumgarten, Flore Nardella, Jiaqi Liang, Samia Miled, Frédéric Bonhomme, Diane Erdmann, Benoit Arcangioli, Paola B. Arimondo, Peter Dedon, Peter Preiser, Artur Scherf

**Affiliations:** a Biology of Host-Parasite Interaction, Institut Pasteur, Paris, France; b CNRS ERL9195, Paris, France; c INSERM U1201, Paris, France; d Antimicrobial Resistance Interdisciplinary Research Group, Singapore-MIT Alliance for Research and Technology, Singapore, Singapore; e Department of Biological Engineering, Massachusetts Institute of Technology, Cambridge, Massachusetts, USA; f School of Biological Sciences, Nanyang Technological University, Singapore, Singapore; g Epigenetic Chemical Biology, Institut Pasteur, UMR 3523, CNRS, France; h Ecole Doctorale MTCI ED563, Université de Paris, Sorbonne Paris Cité, Paris, France; i Université de Paris, CNRS, Institut Jacques Monod, Paris, France; j École technique supérieure du Laboratoire, Paris, France; k Genome Dynamics Unit, Institut Pasteur, UMR 3525, CNRS, Paris, France; National Institute of Allergy and Infectious Diseases

**Keywords:** malaria, tRNA, epitranscriptomic, RNA cytosine methylation, homeostasis, DNMT2, stress response, tRNA cytosine methylation, tRNA modification

## Abstract

Malaria parasites need to cope with changing environmental conditions that require strong countermeasures to ensure pathogen survival in the human and mosquito hosts. The molecular mechanisms that protect Plasmodium falciparum homeostasis during the complex life cycle remain unknown. Here, we identify cytosine methylation of tRNA^Asp (GTC)^ as being critical to maintain stable protein synthesis. Using conditional knockout (KO) of a member of the DNA methyltransferase family, called Pf-DNMT2, RNA bisulfite sequencing demonstrated the selective cytosine methylation of this enzyme of tRNA^Asp (GTC)^ at position C38. Although no growth defect on parasite proliferation was observed, Pf-DNMT2KO parasites showed a selective downregulation of proteins with a GAC codon bias. This resulted in a significant shift in parasite metabolism, priming KO parasites for being more sensitive to various types of stress. Importantly, nutritional stress made tRNA^Asp (GTC)^ sensitive to cleavage by an unknown nuclease and increased gametocyte production (>6-fold). Our study uncovers an epitranscriptomic mechanism that safeguards protein translation and homeostasis of sexual commitment in malaria parasites.

## INTRODUCTION

Malaria remains a major health burden, with more than 400,000 victims every year and 250 million clinical cases (1). The disease is caused by a unicellular parasite species belonging to the *Plasmodium* genus. Plasmodium falciparum, the most pathogenic species, is transmitted to humans via bites from infected *Anopheles* mosquitoes. Malaria pathogens rely on multiple layers of epigenetics in order to regulate gene expression across their complex life cycle (for review, see reference 2). This fast-evolving research field has recently revealed several unprecedented epigenetic mechanisms in P. falciparum such as histone clipping (3), the role of noncoding RNAs in regulating virulence gene expression and sexual commitment (4, 5) as well as the role of the RNA exosome-linked RNase (Rrp6) in ncRNA decay regulation (6). Furthermore, the detection of an unusual DNA cytosine modification has been recently linked to P. falciparum gene expression (7).

Besides epigenetics, RNA modifications that impact gene expression without changing the ribonucleotide sequence are grouped under the term epitranscriptomics (8, 9). The latter is a newly emerging field that is still in its infancy in malaria parasite research. Two recent studies addressed for the first time modifications of ribonucleosides in mRNA (10) and tRNAs (11) in P. falciparum, making extensive use of mass spectrometry (MS). It has been shown that similar to the case with other eukaryotes, tRNAs in P. falciparum can be heavily modified and at least 28 different ribonucleoside modifications, including m^6^A (N6-methyladenosine), m^5^C (5-methylcytosine), and more complex hypermodifications such as mcm^5^U (5-methoxycarbonylmethyluridine) and m^4^Cm (N4,2′-O-dimethylcytidine), have been identified. These modifications are developmentally regulated across the intraerythrocytic life cycle and have the potential to modulate translation efficiency (11). The role of the reversible and dynamic tRNA modifications and their corresponding “writers” are a very promising new research field that may unravel missing regulatory mechanisms of malaria parasite stress and disease factors.

The human malaria parasite P. falciparum belongs to a small group of eukaryotes considered “DNMT2-only organisms” (12). The genome of P. falciparum includes only one single gene annotated as cytosine-5 DNA methyltransferase (*PF3D7_0727300*). However, similar to what is observed in other eukaryotes (for review, see reference 13), the function of Pf-DNMT2 in the malaria parasite is still disputed. Two distinct studies using different Pf-DNMT2 regions expressed as recombinant proteins have come to contradictory conclusions. One study suggested that the recombinant enzyme methylates DNA based on *in vitro* methylation activity assays (14), whereas the other observed methylation activity *in vitro* on tRNA with no activity on DNA substrates (15). However, no functional knockout (KO) of Pf-DNMT2 (Pf-DNMT2KO) has been made that would support any of the proposed roles.

Given the potential role of Pf-DNMT2 in epigenetic gene regulation and epitranscriptome-mediated translational control, we explored the function of this enzyme in P. falciparum asexual blood-stage development. Pf-DNMT2 gene inactivation showed no detectable growth deficit during asexual-stage proliferation under standard *in vitro* culture conditions. Pf-DNMT2 mutant analysis showed that this enzyme acts primarily as a methyltransferase at cytosine 38 of tRNA^Asp (GTC)^ (adjacent to the anticodon loop). C38 tRNA^Asp (GTC)^ mutant parasite analysis revealed the downregulation in a subset of proteins that shifts the parasite metabolism, including the glycolytic pathway. These Pf-DNMT2-dependent changes influence the response to cellular stress. For example, in nutrient-stressed parasites, a drastic decrease in full-length (FL) tRNA^Asp (GTC)^ levels occurred (>70%), possibly by cleavage of an unidentified nuclease. This type of metabolic stress is associated with ap2-g upregulation and high levels of gametocyte production. In addition, mutant parasites show a greater sensitivity to the antimalarial drug dihydroartemisinin than do wild-type 3D7 (3D7-WT) parasites. Altogether, our data support the role of Pf-DNMT2-mediated tRNA cytosine methylation in blood-stage homeostasis by safeguarding malaria protein synthesis during cellular stress.

## RESULTS

### The putative DNA/RNA methyltransferase Pf-DNMT2 is not essential for the intraerythrocytic growth of P. falciparum.

**FIG 1:**
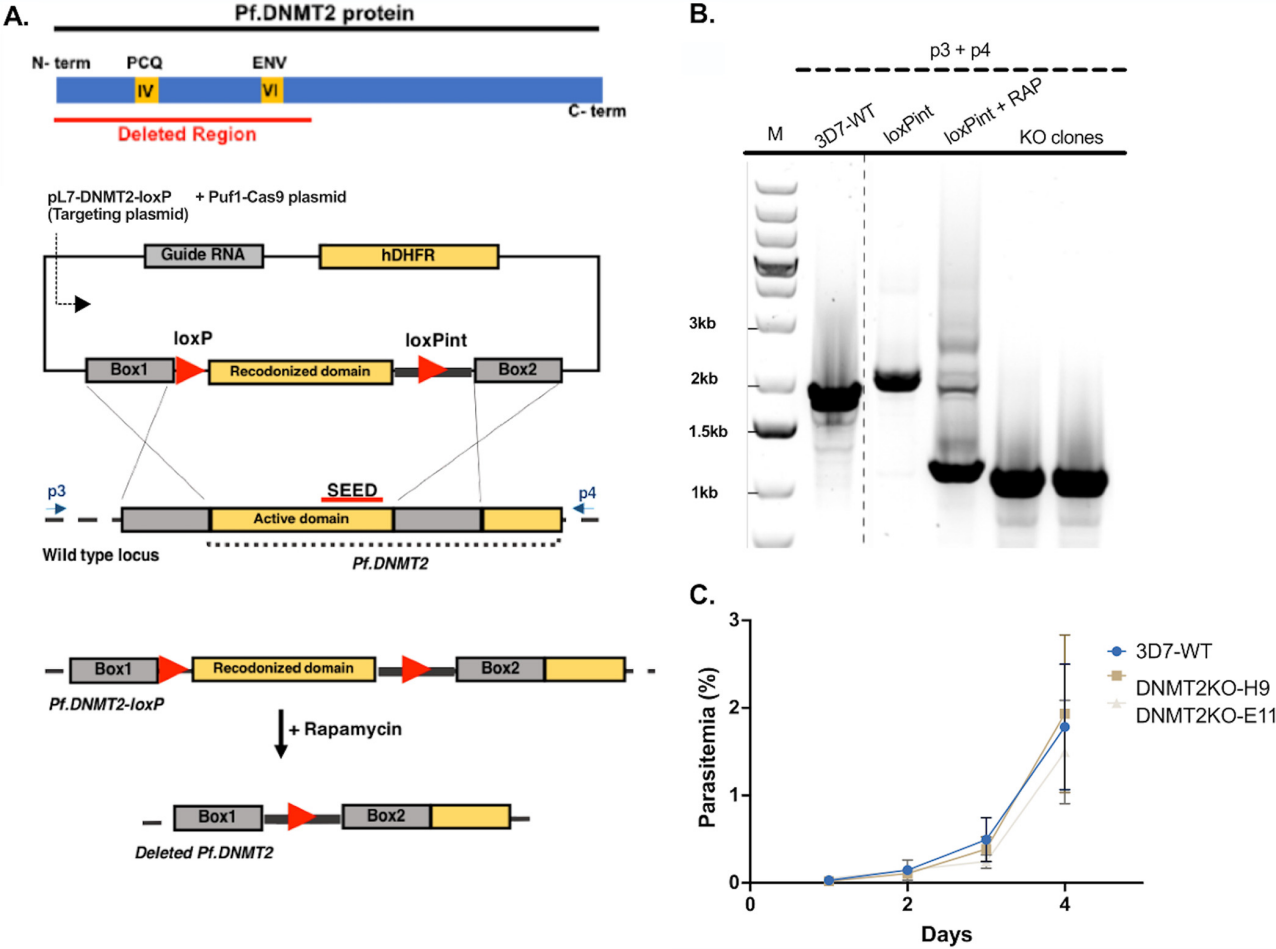
The putative DNA/RNA methyltransferase Pf-DNMT2 is not essential for the intraerythrocytic growth of P. falciparum. (A) (Top) Schematic of Pf-DNMT2; underlined in red is the domain targeted for deletion containing the two conserved predicted DNA methyltransferase motifs IV (PCQ) and VI (ENV) (yellow boxes). (Bottom) Schematic of the conditional knockout strategy. (B) PCR data validating the conditional knockout system. Primers p3 and p4 binding upstream and downstream of homology boxes were used. Expected bands: wild type (WT), 1,995 bp; loxPint before addition of rapamycin (-RAP), 2,135 bp; loxPint after addition of rapamycin (loxPint +RAP) and DNMT2 knockout clones, 1,165 bp. (C) Growth analysis of DNMT2KO clones H9 and E11 and the 3D7-WT control. Means ± SDs are shown (*n* = 3).

Given that malaria parasites belong to a small group of DNMT2-only organisms, we generated a Pf-DNMT2 mutant strain to explore its function in this protozoan pathogen. We applied the previously described loxP-inducible knockout system (16) to delete the two catalytic motifs PCQ and ENV, known to be crucial for the enzyme’s catalytic activity (17) (Fig. 1A). The insertion of two loxP sites and an artificial intron resulted in a shift in the PCR band obtained with primers p3 and p4, from 1,995 bp in 3D7-WT to 2,135 bp in the loxPint transgenic line (Fig. 1B). The addition of rapamycin (RAP) induced the deletion of the region of interest flanked by the two loxP sites, resulting in a lower predominant band at the expected size of 1,165 bp (Fig. 1B). DNA sequence analysis of the obtained PCR product confirmed the correct recombination event. Two clones (DNMT2KO-H9 [H9] and DNMT2KO-E11 [E11]) were isolated from the bulk culture induced by RAP with no trace of intact DNMT2 gene (Fig. 1B). Parasite growth analysis done by measuring DNA content over the course of 4 days showed no significant difference in the asexual growth of DNMT2KO-H9 and -E11 compared to the 3D7-WT control (Fig. 1C). Taken together, our data show that Pf-DNMT2 is dispensable for the asexual intraerythrocytic growth of P. falciparum, which corroborates a previously published data set based on a transposable saturation mutagenesis of P. falciparum (18).

### Pf-DNMT2 is a tRNA cytosine-methyltransferase with a low enzymatic activity on DNA.

**FIG 2:**
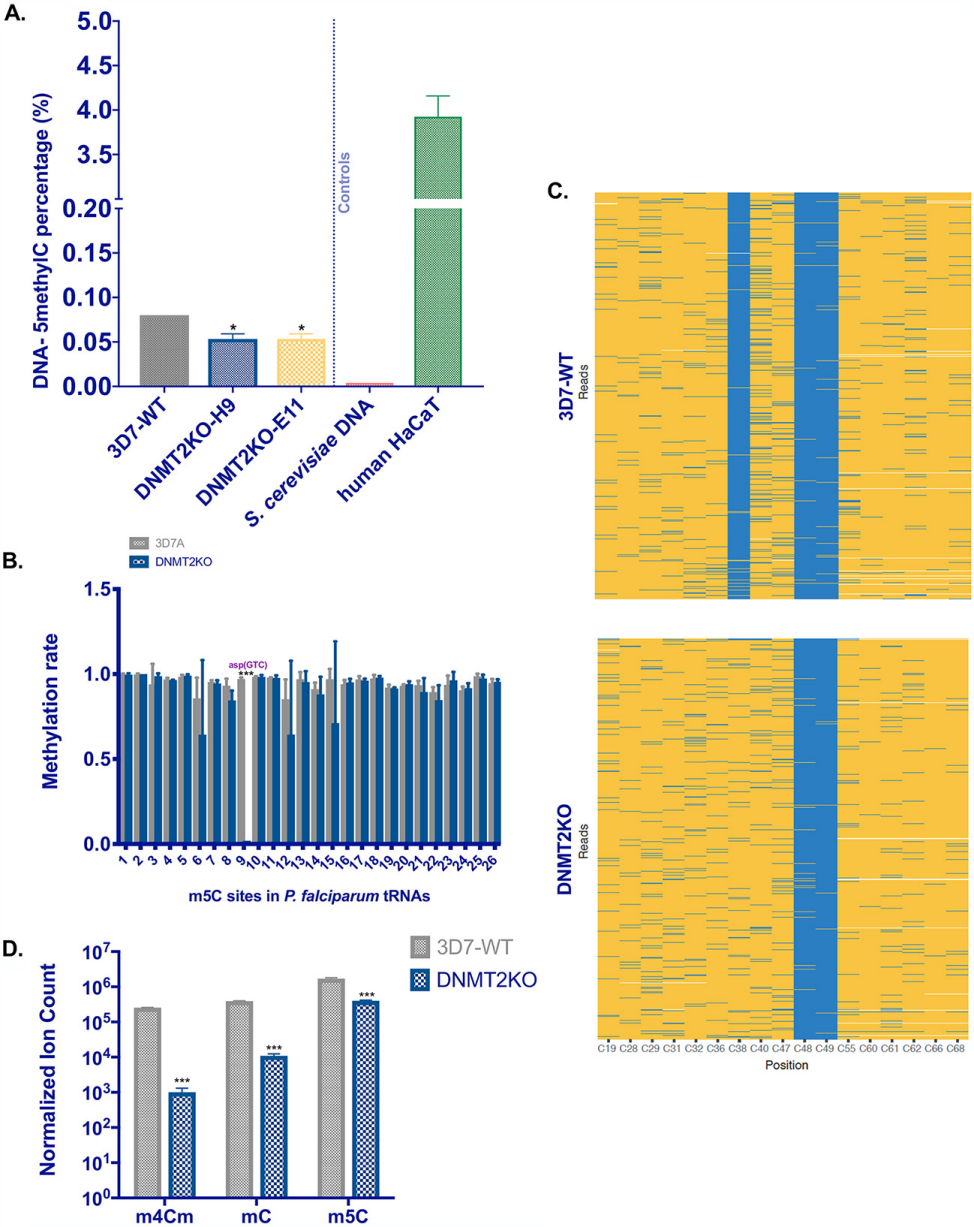
Pf-DNMT2 is a tRNA methyltransferase with a low detectable activity on DNA substrates. (A) LC/MS-MS quantification of 5mdC in gDNA of 3D7-WT and the two DNMT2KO clones H9 and E11. gDNAs of S. cerevisiae and the human keratinocyte HaCaT cell line were used as negative and positive controls, respectively. (B) Column graph representing total methylated cytosine (m5C) sites in bulk tRNA, identified using RNA bisulfite sequencing, with their corresponding methylation rates in 3D7-WT and DNMT2KO strains. (C) Heat map showing the methylation status of all cytosine positions in aspartic acid (GTC) tRNA, as determined by RNA-BS-seq in wild-type 3D7 (upper portion) and DNMT2KO (lower portion). Each row represents one sequence read. Blue lines indicate methylated cytosines and yellow lines indicate unmethylated cytosines. (D) Mass spectrometry-based quantification of m5C, m3C, and m4Cm cytosine modifications in tRNA preparations from 3D7-WT or DNMT2KO. Data are presented in normalized ion counts for each cytosine modification. Means ± SDs are shown. (A) *n* = 2; (B and C) *n* = 4 and (D) *n* = 3. ns, nonsignificant. *, *P* < 0.05; **, *P* < 0.01; ***, *P* < 0.001 (unpaired two-tailed *t* tests).

Two previous studies using truncated recombinant Pf-DNMT2 and *in vitro* methyltransferase assays came to controversial conclusions (14, 15). To investigate the biological function of Pf-DNMT2, we developed assays to explore our mutant clones H9 and E11. We first assessed Pf-DNMT2 activity on DNA using liquid chromatography-tandem mass spectrometry (LC/MS-MS). We included two independent biological replicates of the 3D7-WT control strain and each of the two DNMT2KO clones, H9 and E11. In parallel, DNA from the yeast Saccharomyces cerevisiae, known to be devoid of DNA methylation (19), and DNA from human keratinocyte cell line HaCaT were used as negative and positive controls, respectively. The DNA mass spectrometry data show a 30% decrease in the global levels of 5-methyldeoxycytidine (5mdC) in the two DNMT2KO clones (0.055%) compared to the 3D7-WT (0.08%) strain (*P* = 0.0153) (Fig. 2A and Fig. S1). In addition to LC/MS-MS, enzyme-linked immunosorbent assay (ELISA)-based 5mdC detection in genomic DNA (gDNA) gave similar reductions in 5mdC in the two KO clones (Fig. S2).

10.1128/mBio.02558-21.2 FIG S1 LC/MS-MS analysis of P. falciparum WT and DNMT2KO genomic DNA. (a) Chromatogram of a typical injection of digested DNA from 3D7-WT (top) and DNMT2KO (bottom), showing deoxycytidine (dC), deoxyadenosine (dA), deoxyguanosine (dG), and thymidine (dT). Extracted fragment ion chromatogram. (b) dC and (c) 5-methyldeoxycytidine (5mdC). RT, retention time. Download FIG S1, PDF file, 0.4 MB. Copyright © 2021 Hammam et al. 2021 Hammam et al. https://creativecommons.org/licenses/by/4.0/ This content is distributed under the terms of the Creative Commons Attribution 4.0 International license.

10.1128/mBio.02558-21.3 FIG S2 ELISA-based quantification of 5mdC in gDNA of 3D7-WT and DNMT2KO clones H9 and E11. Parasites were synchronized at the schizont stage and gDNA was prepared from three independent biological replicates of each strain (3D7-WT, DNMT2KO-H9, and DNMT2KO-E11). Methylated DNA oligonucleotides were used as positive controls. Download FIG S2, PDF file, 0.05 MB. Copyright © 2021 Hammam et al. 2021 Hammam et al. https://creativecommons.org/licenses/by/4.0/ This content is distributed under the terms of the Creative Commons Attribution 4.0 International license.

To explore the role of Pf-DNMT2 in tRNA methylation, we first used RNA bisulfite sequencing (RNA-BS-seq), the gold standard technique in mapping m^5^C at the base resolution level in RNA (20). Given the previous findings in other eukaryotic organisms showing the implication of DNMT2 in cytosine methylation of tRNAs (21), we carried out RNA-BS-seq on purified tRNAs from DNMT2 mutants and the 3D7-WT control. Based on our robust methylation calling cutoffs, we identified 26 independent m^5^C sites in different tRNA subtypes in the 3D7-WT control (tRNAs delivering 10 distinct amino acids). These include glutamine, alanine, glutamic acid, leucine, aspartic acid, serine, valine, glycine, methionine, proline, and cysteine tRNAs (see Table S1 for details on the detected m^5^C positions). Out of those 26 methylation sites, only one m^5^C site in aspartic acid tRNA, Asp (GTC), showed a drastic reduction in methylation rate in DNMT2 mutant parasites (Fig. 2B). We next zoomed in to the base resolution level in the tRNA^Asp (GTC)^ sequence. The C38 position was devoid of any detectable cytosine methylation in Pf-DNMT2KO (Fig. 2C). The same C38 methylation of tRNA^Asp (GTC)^ was recently identified as a target of a recombinant Pf-DNMT2 (15).

10.1128/mBio.02558-21.8 TABLE S1 m5C positions detected in P. falciparum tRNA using RNA-BS-seq. Download Table S1, XLSX file, 0.02 MB. Copyright © 2021 Hammam et al. 2021 Hammam et al. https://creativecommons.org/licenses/by/4.0/ This content is distributed under the terms of the Creative Commons Attribution 4.0 International license.

Interestingly, methylation of C48 and C49 in this tRNA was maintained in parasites lacking DNMT2 (Fig. 2C). This is in agreement with previous findings from other organisms, showing that these two sites are dependent on another m^5^C tRNA-methyltransferase belonging to the Nsun2 family (22, 23). A putative SUN-like RNA methyltransferase (*PF3D7_1230600*) is described in PlasmoDB (www.PlasmoDB.org) and may be responsible for function in malaria parasites.

In addition to RNA-BS-seq, we adopted a mass spectrometry-based approach to quantify the levels of tRNA cytosine methylation in tRNA isolated from DNMT2 mutants and the 3D7-WT control. Digested tRNA was injected into an Agilent 6490 triple quadrupole mass spectrometer to analyze ribonucleoside modifications. Our mass spectrometry-based quantification showed reduced m^5^C signals (4.25-fold) in parasites lacking Pf-DNMT2 (Fig. 2D). This observation is consistent with the predicted function of Pf-DNMT2 in m^5^C tRNA methylation and corroborates our RNA-BS-seq data (Fig. 2B and C). Unexpectedly, our MS data also showed a striking decrease in other tRNA cytosine modifications in DNMT2 mutants including mC (m^3^C or m^4^C) and the hypermodification m^4^Cm (Fig. 2D). This observation is unprecedented given the fact that DNMT2 in other eukaryotes has been so far only linked with methylation of very specific cytosine residues in few tRNA subsets (21, 24).

We generated a parasite line expressing an episomal 3× hemagglutinin (3HA)-tagged Pf-DNMT2 to study its cellular localization in asexual blood-stage parasites. Western blot and immunofluorescence experiments showed the presence of Pf-DNMT2 in the nucleus and the cytoplasm of P. falciparum (Fig. S3). Likewise, a dual cellular location of DNMT2 in *Drosophila* was observed (25) and may be reflective of complex activity of Pf-DNMT2. Taken together, our data show that Pf-DNMT2 is a tRNA cytosine methyltransferase with selective activity toward C38 in the aspartic acid (GTC) tRNA.

10.1128/mBio.02558-21.4 FIG S3 Episomal expression of Pf-DNMT2 reveals its dual cytoplasmic and nuclear subcellular localization in P. falciparum. (A) Schematic of pln-DNMT2-3HA episome. P cam, Pf-calmodulin promoter; 3HA, 3×HA tag sequences; UTR, untranslated region; BSD, blasticidin selectable marker. (B) Western blot analysis of Pf-DNMT2 expression in the cytoplasmic and nuclear fractions of synchronous rings and trophozoite and schizont stage parasites. Anti-HA antibodies were used to detect Pf-DNMT2 expression (expected size at 82 kDa). Anti-Pf-histone H3 and Pf-aldolase antibodies were used as nuclear and cytoplasmic controls, respectively. CE, cytoplasmic extracts; NE, nuclear extracts. (C) Immunofluorescence assays using anti-HA antibodies. DAPI, nuclear markers; BF, bright field. Scale bar = 5 μm. Download FIG S3, PDF file, 0.6 MB. Copyright © 2021 Hammam et al. 2021 Hammam et al. https://creativecommons.org/licenses/by/4.0/ This content is distributed under the terms of the Creative Commons Attribution 4.0 International license.

### Loss of DNMT2-mediated tRNA^Asp (GTC)^ C38 methylation is associated with the downregulation of proteins with Asp GAC codon bias.

**FIG 3:**
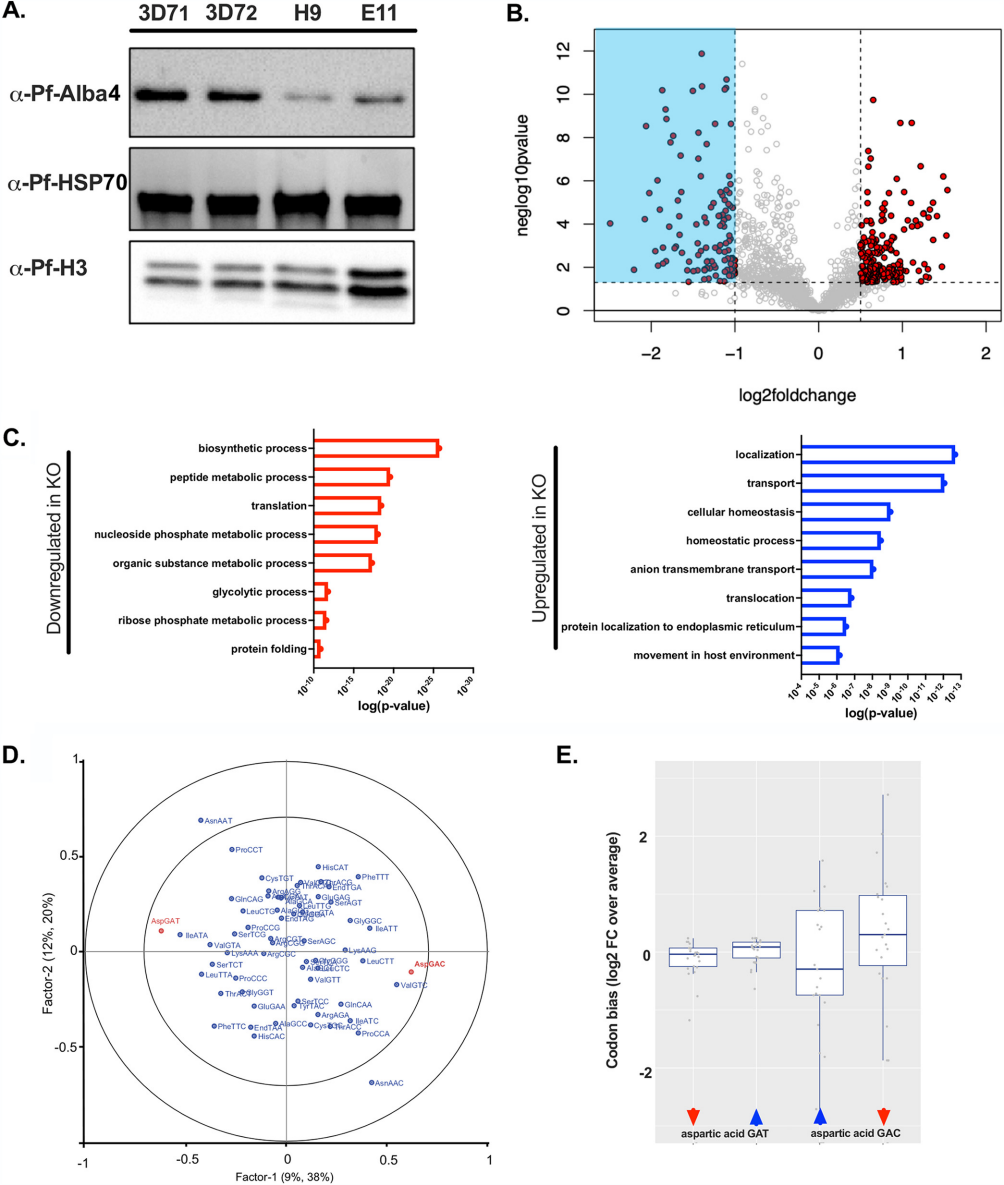
Loss of DNMT2-mediated tRNA Asp (GTC) C38 methylation is associated with the downregulation of proteins with Asp GAC codon bias. (A) Western blot analysis of Pf-Alba4 expression in 3D7 and DNMT2 mutant. HSP70 and histone H3 antibodies were used as negative controls. (B) Volcano plot illustrating the differentially expressed proteins detected by MS in DNMT2 mutants compared to 3D7-WT. (C) GO enrichment analysis of the top down- and upregulated proteins in DNMT2KO compared to 3D7-WT. (D) Partial least-squares regression (PLS-R) analysis of the codon bias in differentially expressed genes in DNMT2KO versus 3D7-WT. (E) Boxplots representing the Asp GAT and Asp GAC codon biases in downregulated (red arrows) and upregulated proteins (blue arrows) in DNMT2 mutants. (Statistical significance was calculated using paired two-tailed t tests.)

Since cytosine methylation in tRNAs has been previously linked to protein synthesis (23, 26), we first conducted an *in silico* bioinformatic identification of proteins with Asp GAC codon bias (Table S2). We focused on proteins expressed during the asexual blood stages of the parasite cycle and selected the top hits based on the total number of Asp GAC codons they contain. Among these candidates, several chaperones and heat shock proteins are present as well as different kinases. One of the top candidates is the DNA/RNA binding protein Pf-Alba4 (*PF3D7_1347500*), with a count of 6 Asp GAC codons (1.6 Asp GAC codons/100 amino acids [aa]). To gain insight into the effect of the loss of tRNA^Asp (GTC)^ C38 methylation on protein translation, we analyzed by Western blotting the expression of Pf-Alba4 in total extracts from Pf-DNMT2 mutants and the 3D7-WT control. Our Western blot data show a significant decrease in Pf-Alba4 expression in the mutants compared to the 3D7-WT control (Fig. 3A). The expressions of two control proteins (Pf-HSP70 and Pf-histone 3), both lacking aspartic acid GAC codons, remained unchanged (Fig. 3A).

10.1128/mBio.02558-21.9 TABLE S2 List of proteins candidates with a GAC codon bias expressed in P. falciparum asexual blood stages. Download Table S2, XLSX file, 0.08 MB. Copyright © 2021 Hammam et al. 2021 Hammam et al. https://creativecommons.org/licenses/by/4.0/ This content is distributed under the terms of the Creative Commons Attribution 4.0 International license.

Based on this result, we carried out a quantitative proteomics study. Tandem mass tag (TMT)-based quantitative proteomics of trophozoites in the wild type (3D7) and knockout (Pf-DNMT2KO) resulted in robust identification and quantitation of 1,400 to 1,500 proteins in every sample, covering 27.5% of the proteome. Analysis of fold change data (3D7/Pf-DNMT2KO) revealed the following numbers of significantly altered proteins (log_2_ fold change greater than 0.5 or less than 1 relative to DNMT2KO, *P* < 0.05): 199 proteins were enriched in the WT cells and 114 proteins were enriched in the DNMT2KO cell lines (Fig. 3B and Table S3). Gene ontology (GO) enrichment analysis of the top downregulated proteins in DNMT2 mutants indicates that these proteins are mainly involved in different metabolic processes, such as the glycolytic pathway as well as protein translation and folding. On the other hand, upregulated proteins are mainly involved in protein transport, homeostasis, and movement within the host (Fig. 3C). It is noteworthy that proteins downregulated in Pf-DNMT2 mutants showed increased usage for the Asp GAC codon compared to upregulated proteins (*P* = 0.09) (Fig. 3D and E), whereas proteins using the second Asp codon (GAT) showed a rather unbiased profile (*P* = 0.12) (Fig. 3D and E). Altogether, our data strongly suggest that the absence of tRNA^Asp (GTC)^ C38 methylation is directly correlated with the downregulation of proteins with aspartic acid GAC codon bias.

10.1128/mBio.02558-21.10 TABLE S3 Proteomics data. Download Table S3, XLSX file, 0.8 MB. Copyright © 2021 Hammam et al. 2021 Hammam et al. https://creativecommons.org/licenses/by/4.0/ This content is distributed under the terms of the Creative Commons Attribution 4.0 International license.

### Loss of Pf-DNMT2 increases stress-induced sexual commitment and sensitivity to dihydroartemisinin (DHA) in malaria parasites.

**FIG 4:**
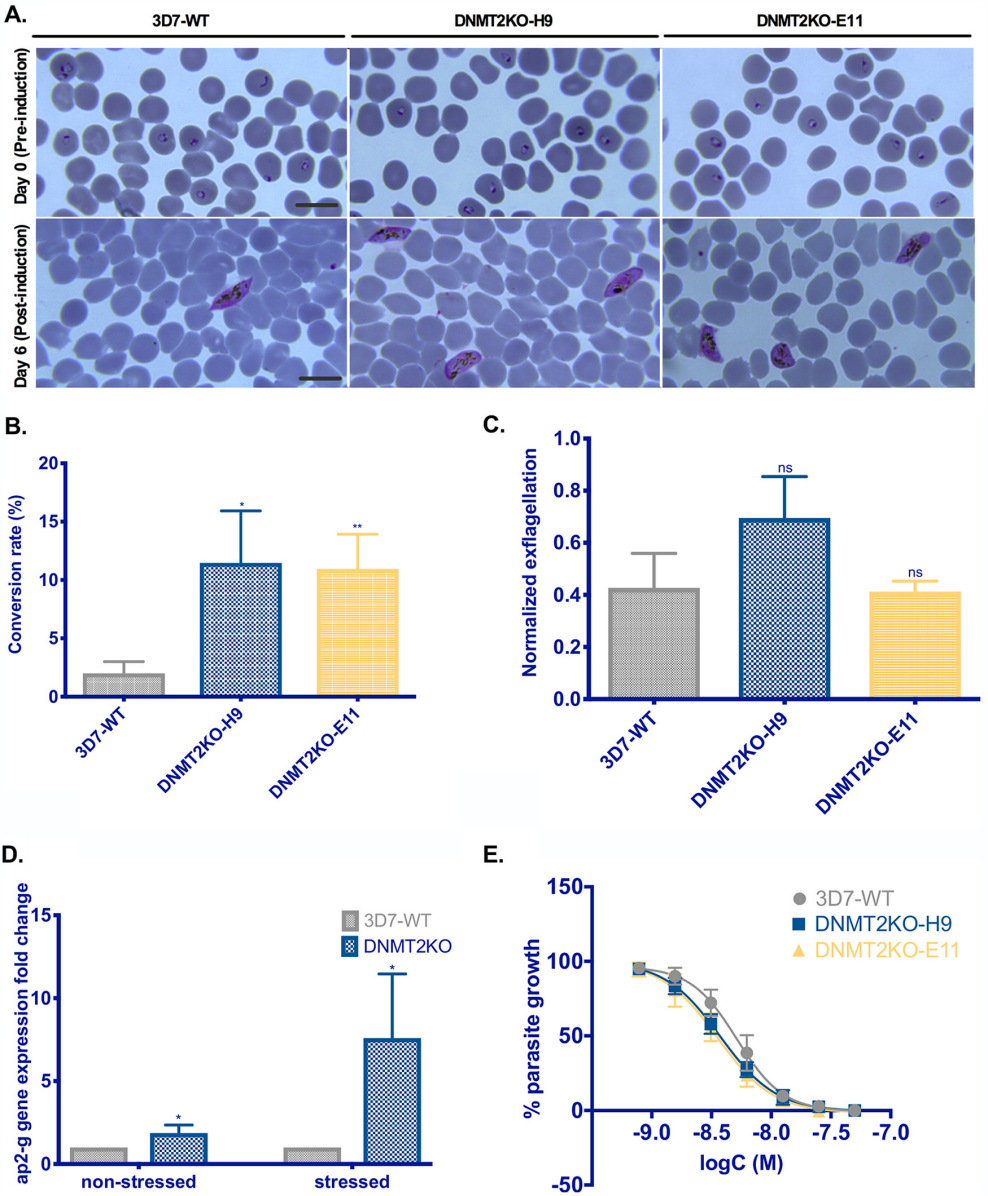
Loss of Pf-DNMT2 increases stress-induced sexual commitment and greater sensitivity to DHA in malaria parasites. (A) Giemsa-stained thin blood smears of wild-type 3D7 and two DNMT2KO clones (DNMT2KO-H9 and DNMT2KO-E11) on day 0 (top, committed rings) and on day 6 after gametocytes induction (bottom, stage III gametocytes). Scale bar = 10 μm. (B) Gametocyte conversion rates. Conversion rates are equal to gametocytemia (percent) counted on day 6 divided by parasitemia in rings (percent) counted on day 0 multiplied by 100. (C) Normalized *in vitro* exflagellation. The number of exflagellation centers is normalized to the corresponding gametocytemia at stage V. (D) qPCR quantification of ap2-g gene expression. Data are presented as the gene expression fold change relative to the 3D7 control. (E) Survival curves obtained after 72 h of dihydroartemisinin (DHA) treatment. Parasite growth is plotted in function of DHA concentration (logC in molar concentration). Means ± SDs are shown. (B) *n* = 3; (C) *n* = 2; (D) *n* = 4; (E) *n* = 3. *, *P* < 0.05; **, *P* < 0.01 (unpaired two-tailed *t* tests). Data are from 1 (D), 2 (C), or 3 (B and E) independent experiments.

Since this pathogen encounters numerous environmental changes during the life cycle progression in the human and mosquito hosts, we investigated whether the observed changes in the proteome in Pf-DNMT2KO render mutant parasites more sensitive to cellular stress. To this end, we induced gametocytogenesis in Pf-DNMT2KO clones H9 and E11 along with the parental 3D7A DiCre strain (3D7-WT) using a previously established nutrient depletion protocol (27). This protocol relies on metabolic stress-induced gametocytogenesis by reusing parasite-conditioned medium for several days. Both Pf-DNMT2KO parasite clones showed a 6-fold increase in the gametocyte conversion rate (CR; average CR =12%) compared to the control strain 3D7-WT, for which the average CR was around 2% (*P* [DNMT2KO-H9] = 0.023; *P* [DNMT2KO-E11] = 0.0078) (Fig. 4B). Episomal expression of the full-length (FL) DNMT2 under the control of the calmodulin promoter in the KO background was able to partially restore the gametocyte conversion rate (33% decrease) (Fig. S4). Giemsa-stained smears of cultures on day 0 (preinduction) showed mostly the presence of synchronous rings in all three strains 3D7-WT, DNMT2KO-H9, and DNMT2KO-E11 (Fig. 4A, upper portion). On day 6 after gametocyte induction, Giemsa-stained smears of parasite cultures showed the presence of highly synchronous stage III gametocytes, with no morphological differences observed between WT and KO strains (Fig. 4A, lower portion). We next checked if mature gametocyte stage V lacking Pf-DNMT2 is able to exflagellate (conversion into male gametes by lowering the temperature to 26°C). Our *in vitro* exflagellation data showed no detectable difference in exflagellation rates between gametocytes lacking Pf-DNMT2 and the 3D7-WT control, when normalized to the gametocytemia (*P*= 0.89) (Fig. 4C). Finally, we quantified the gene expression of ap2-g, the master regulator of sexual commitment in P. falciparum, under gametocyte induction culture conditions for Pf-DNMT2 mutants and 3D7-WT (28–30). Reverse transcription-quantitative PCR (qRT-PCR) quantification on highly synchronous stressed cultures showed up to 5-fold upregulation of ap2-g in Pf-DNMT2KO clones compared to the 3D7-WT strain (*P* = 0.0358) (Fig. 4D). Interestingly, a 2-fold increase in ap2-g expression was observed in Pf-DNMT2KO parasites under normal culture conditions, compared to their 3D7-WT counterparts (*P* = 0.042) (Fig. 4D). Taken together, our data strongly suggest that the loss of Pf-DNMT2 destabilizes the parasite asexual blood-stage homeostasis and “primes” the parasites to being more sensitive to metabolic stress situations. The downstream events of gametocyte development and exflagellation were seemingly not affected in our *in vitro* culture assays.

10.1128/mBio.02558-21.5 FIG S4 Episomal expression of Pf-DNMT2 partially restores the gametocyte conversion rate in the DNMT2KO strain. Full-length DNMT2 was expressed in one of the two DNMT2KO clones (rescued KO) and gametocyte induction was carried out *in vitro* in the 3D7, KO clone H9, and the rescued KO, as described earlier. Gametocyte conversion rate is calculated by dividing the gametocytemia (calculated using FACS double staining with SYBR green and Mitotracker) on day 6 after induction by the parasitemia in rings on day 0 preinduction and multiplying by 100. Statistical analysis was conducted using unpaired two tailed *t* tests. *, *P* < 0.05. Download FIG S4, PDF file, 0.04 MB. Copyright © 2021 Hammam et al. 2021 Hammam et al. https://creativecommons.org/licenses/by/4.0/ This content is distributed under the terms of the Creative Commons Attribution 4.0 International license.

Given the observed metabolic shift and higher sensitivity to stress response of Pf-DNMT2KO parasites, we investigated their susceptibility to the current first-line treatment, dihydroartemisinin (DHA) (for review, see reference 31). Our data show a shift toward lower concentration values in the survival curves of Pf-DNMT2KO clones H9 and E11 than for the 3D7-WT control, indicating an increased sensitivity to DHA (Fig. 4E). The calculated mean 50% inhibitory concentration (IC_50_) of DHA (±standard deviation [SD]) was 5.1 nM (±0.909) in 3D7-WT (95% confidence interval [CI]: 4.715 nM to 5.62 nM) and dropped to 3.7 nM (±0.63) (95% CI: 3.41 nM to 4.04 nM) and 3.5 nM (±0.935) (95% CI: 3 nM to 9.98 nM) in DNMT2KO-H9 and DNMT2KO-E11, respectively. Altogether, our data show that depletion of Pf-DNMT2 creates a cellular state that predisposes mutant parasites to stress situations as illustrated in Fig. 4.

### Metabolic stress induces tRNA^Asp (GTC)^ degradation and the downregulation of aspartic acid GAC-biased proteins in DNMT2 mutants.

In order to investigate how metabolic stress affects protein translation in the Pf-DNMT2 mutants, we investigated parasite tRNA^Asp (GTC)^ stability and proteome from nutrient-stressed 3D7-WT and Pf-DNMT2KO parasites using the conditions described earlier to induce sexual commitment.

**FIG 5:**
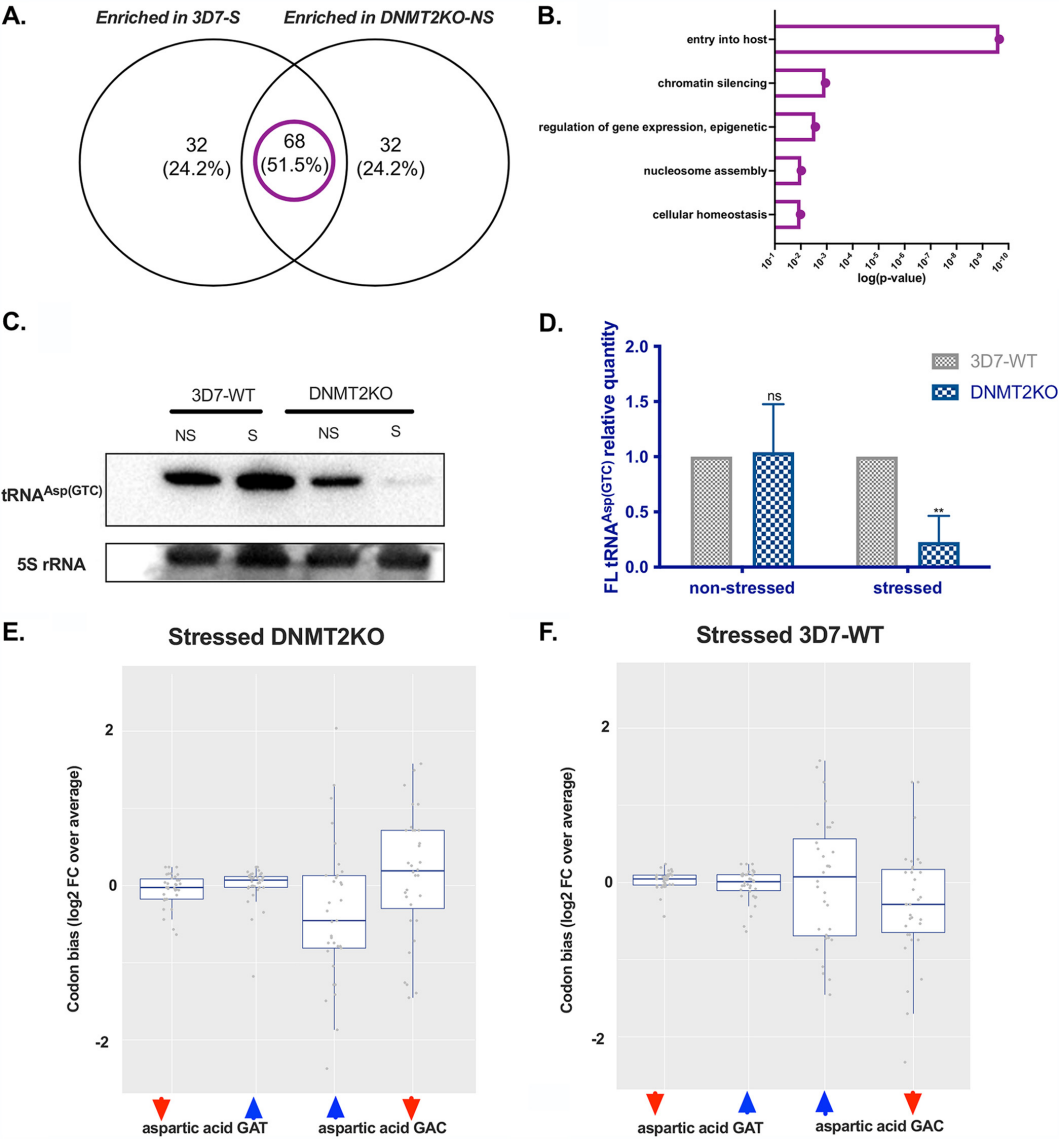
Metabolic stress induces tRNA Asp (GTC) degradation and the downregulation of aspartic acid GAC-biased proteins in DNMT2 mutants. (A) Venn diagram showing the percent overlap between proteins enriched in 3D7 stressed and KO nonstressed organisms. (B) GO enrichment analysis of the common enriched proteins between 3D7 stressed and KO nonstressed organisms. (C) Northern blot analysis using a specific 3′-tRNA^Asp (GTC)^ biotinylated probe on total RNA of 3D7-WT and DNMT2KO strains from either nonstressed (NS) or stressed (S) conditions. The 5S rRNA probe was used as an internal loading control. The Northern blot shown is representative of three different replicates. (D) Relative quantification of full-length (FL) tRNA^Asp (GTC)^ band detected in the Northern blot of panel A. (E) Boxplots representing the Asp GAT and Asp GAC codon biases in up (blue arrows) and downregulated (red arrows) proteins in stressed DNMT2 mutants. (F) Boxplots representing the Asp GAT and Asp GAC codon biases in up (blue arrows) and downregulated (red arrows) proteins in stressed 3D7-WT. Means ± SDs are shown. (C and D) *n* = 3 and *n* = 2 to 4. *, *P* < 0.05; **, *P* < 0.01 (unpaired two-tailed *t* tests). Panels E and F represent paired two-sample *t* tests).

We first explored whether the loss of C38 methylation of tRNA^Asp (GTC)^ impacts tRNA stability under nutritional stress. To this end, we extracted total RNA from nonstressed and metabolically stressed Pf-DNMT2 mutants and 3D7-WT control parasites. tRNA levels were analyzed using Northern blotting to assess the levels of FL mature tRNA^Asp (GTC)^ as well as the presence of tRNA fragments arising from potential tRNA cleavage. Our Northern blots using a specific 3′-end Asp (GTC) probe showed a significant decrease in the level of FL tRNA^Asp (GTC)^ (at around 75 bp) isolated from mutant parasites compared to the 3D7-WT control (Fig. 5C). Relative quantification of the FL tRNA band intensities from three independent biological replicates is shown in Fig. 5D. Our quantification suggests up to 75% decrease in FL tRNA^Asp (GTC)^ in stressed Pf-DNMT2 mutants (*P* = 0.0048) (Fig. 5D). In addition, shorter tRNA bands (around 20 to 30bp) were detectable in overexposed RNA blots for DNMT2 mutants but not for the 3D7-WT control, suggesting that this type of stress induces tRNA^Asp (GTC)^ processing by a nuclease leading to the observed low tRNA^Asp (GTC)^ levels (Fig. S5). Importantly, episomal expression of the full-length Pf-DNMT2 gene in one of the knockout clones (Pf-DNMT2-rescued KO) restored the wild-type phenotype in terms of FL-tRNAs abundance and absence of shorter tRNA fragments (Fig. S5). In addition, we also used Northern blotting to investigate whether the expression of other tRNAs was affected in the DNMT2 mutants. We randomly picked two additional tRNAs (arginine and valine) along with the aspartic acid tRNA. Our Northern blot analysis shows a decrease in the global levels of Arg and Val tRNAs in the stressed DNMT2KO compared to the 3D7-WT (2.7- and 4.6-fold decreases, respectively). Our data suggest that tRNA^Asp (GTC)^ is the most unstable in the stressed DNMT2KO, with a 20-fold decrease compared to other tRNAs (Fig. S6).

10.1128/mBio.02558-21.6 FIG S5 tRNA fragments are detectable in stressed DNMT2 mutants. Shown is Northern blot analysis of FL-tRNA^Asp (GTC)^ abundance (top) and tRNA fragments (bottom) in DNMT2 mutants, 3D7-WT, and rescued DNMT2KO. Rescued DNMT2KO, DNMT2KO clone H9 transfected with the episome expressing full-length DNMT2; 3D7-WT, 3D7 wild type; E11 and H9, the two mutant clones. Red arrows indicate tRNA fragments at around 30 and 45 bp. RNA ladder: DynaMarker Prestain Marker for Small RNA Plus. Download FIG S5, PDF file, 0.09 MB. Copyright © 2021 Hammam et al. 2021 Hammam et al. https://creativecommons.org/licenses/by/4.0/ This content is distributed under the terms of the Creative Commons Attribution 4.0 International license.

10.1128/mBio.02558-21.7 FIG S6 Northern blot analysis of the abundance of tRNAs in DNMT2 mutant versus 3D7-WT. The left portion shows the Northern blots for the expression of aspartic acid (Asp), arginine (Arg), and alanine (Ala) tRNAs in 3D7 wild type and the DNMT2KO parasites, under both nonstressed (NS) and stressed (S) conditions. 5S rRNA was used as the RNA loading control. The right portion shows the relative quantification of the corresponding tRNA bands. Download FIG S6, PDF file, 0.2 MB. Copyright © 2021 Hammam et al. 2021 Hammam et al. https://creativecommons.org/licenses/by/4.0/ This content is distributed under the terms of the Creative Commons Attribution 4.0 International license.

Proteome analysis revealed an unexpected but important observation. Stressed 3D7-WT parasites shared up to 51.5% of their upregulated proteins with nonstressed Pf-DNMT2KO parasites (Fig. 5A). Shared proteins are mostly involved in gene expression regulation and cellular homeostasis (Fig. 5B). This observation is intriguing and strongly supports the notion that the Pf-DNMT2 mutants, grown under normal culture conditions, are already primed for being more susceptible to stressful environments. This corroborates the 2-fold upregulation of ap2-g in nonstressed DNMT2 mutants depicted in Fig. 4D.

The highly reduced stability of tRNA^Asp (GTC)^ in stressed DNMT2 mutants pointed toward a potential downstream effect on the translation of proteins with Asp GAC codon bias. Similar to the case for Fig. 3C, we calculated the changes of the codon frequencies in the upregulated and downregulated proteins in stressed DNMT2 mutants (Fig. 5E). This again revealed the expected significant bias for the Asp GAC codon among the downregulated proteins compared to their upregulated counterparts (*P* = 0.028). The opposite tendency was observed in stressed 3D7-WT, for which our data show an underrepresentation of Asp GAC in downregulated proteins (Fig. 5F). In addition, no differences in Asp GAT codon frequencies were observed in either stressed Pf-DNMT2 mutants or stressed 3D7-WT (Fig. 5E and F). Altogether, the data from Northern blot analysis and mass spectrometry-based protein quantification strongly suggest that the loss of DNMT2-mediated C38 methylation in tRNA^Asp (GTC)^ renders this specific tRNA very unstable in the presence of nutrient depletion stress, which triggers its cleavage and the subsequent downregulation of proteins with aspartic acid GAC codon bias. This is a well-established paradigm in model organisms (32). The downregulation of GAC codon-biased proteins destabilizes the parasite’s homoeostasis, which results in an increase in sexual commitment.

## DISCUSSION

Dynamic changes in tRNA modifications have been linked to the control of protein translation in P. falciparum (11), indicating that an emerging level of regulation lies in the epitranscriptomic modification of tRNAs. In this work, we identify a specific tRNA cytosine methylation site that impacts expression of a subset of codon-biased proteins and safeguards malaria parasite homeostasis to various types of cellular stress. Writers of RNA cytosine modifications and their biological role for this pathogen are still ill defined. Together with Drosophila melanogaster, Dictyostelium discoideum, Entamoeba histolytica, and Schizosaccharomyces pombe, P. falciparum belongs to a small group of “DNMT2-only” organisms, as they do not encode any other enzymes of the DNMT family that are known DNA methyltransferase enzymes. The function of DNMT2 as a DNA and/or RNA methyltransferase in DNMT2-only organisms, including P. falciparum, is still debated (12, 14, 15).

In this work, we generated a Pf-DNMT2 gene KO to investigate its role in cytosine methylation of plasmodial RNA and DNA. Similar to findings for other eukaryotes (33), mutant Pf-DNMT2 parasites are viable and show no growth defect in asexual blood-stage proliferation under standard *in vitro* culture conditions. LC/MS-MS 5mdC quantification in parasite gDNA showed a reduction in the global DNA methylation levels in parasites lacking DNMT2 (0.053%) compared to 3D7-WT (0.08%). This observation may indicate a very low activity of the enzyme on DNA methylation, as suggested by a previous report for *Plasmodium* (14) and reports for other DNMT2-only organisms (34, 35). Given that the global DNA methylation levels in P. falciparum are in general very low compared to those in higher eukaryotes, we cannot exclude that the observed changes of DNA methylation in the KO parasites could result from human DNA contamination. Although we used a filtration purification procedure of human red blood cells (RBC) that eliminates white blood cells (WBC), our fluorescence-activated cell sorting (FACS) analysis of the RBC source used for P. falciparum culture still detected the presence of very low levels of human WBC.

Based on this finding, we followed up on the observation earlier, namely, that DNMT2 is a tRNA methyltransferase (15, 21). We combined RNA bisulfite sequencing with mass spectrometry analysis to investigate the effect on parasites lacking Pf-DNMT2 activity. In P. falciparum, we show that Pf-DNMT2 methylates exclusively tRNA^Asp (GTC)^ at position C38. Two other cytosine methylation sites (positions C48 and C49) in tRNA^Asp (GTC)^ were not affected in Pf-DNMT2 mutant parasites. However, mass spectrometry of purified tRNA revealed a more multifaceted picture. It confirmed that m5C methylation in bulk tRNAs is reduced and, unexpectedly, also showed that other cytosine methylation modifications (mC and m4Cm) were reduced in the total tRNA fraction compared to those in 3D7. These two modifications are not able to be detected by the bisulfite sequencing technique. Thus, by combining two techniques, we observed that the plasmodial Pf-DNMT2 enzyme may have a wider spectrum of cytosine methylation modifications than those reported for other organisms based on RNA bisulfite sequencing only. However, we cannot exclude alternative explanations. There are several possible explanations for the link between Pf-DNMT2-mediated m5C and the levels of m3C/m4C and m4Cm in tRNA. One is that m5C serves as a “checkpoint” tRNA modification that must be in place before other modifications (e.g., m3C/m4C and m4Cm) can be inserted. There are numerous examples of this behavior (36), including the requirement for s2U at position 33 of the single tRNA for Trp in Trypanosoma brucei for C deamination editing of the anticodon from CCA to UCA to allow reading of the mitochondrial UGA codon (37). Another m5C-dependent mechanism involves protection against enzymatic cleavage of tRNAs also containing m3C/m4C and m4Cm. NSUN2-mediated m5C protects tRNA from cleavage by angiogenin in human cells (38), similarly to DNMT-mediated m5C with *Drosophila* tRNA (32). If m5C38-containing tRNAs also contain m3C/m4C and m4Cm, then tRNA degradation due to m5C-dependent cleavage will concomitantly reduce m3C/m4C and m4Cm. Alternatively, Pf-DNMT2-mediated changes in other modifications may be due to not-yet-detected altered cellular phenotypes in Pf-DNMT2 mutant parasites. Altogether, our KO data show that Pf-DNMT2 is primarily a tRNA cytosine methyltransferase. Our mass spectrometry analysis and published data on a recombinant Pf-DNMT2 protein (14) suggest that this enzyme has a low methyltransferase activity on DNA. This corroborates our data showing the dual subcellular localization of DNMT2 in both the nucleus and cytoplasm of the parasite (Fig. S3).

Although parasites lacking Pf-DNMT2 did not reveal any obvious growth phenotype using standard *in vitro* culture condition for asexual blood stages, intriguingly, Pf-DNMT2KO showed significant changes in protein expression. Proteins biased for GAC codons were significantly downregulated compared to the case with wild-type parasites. Among the upregulated proteins, proteins with GAC codons were underrepresented. Proteins whose synthesis depends on the second aspartic acid codon GAT did not show this bias, corroborating a role for Pf-DNMT2 in the expression of a specific subset of proteins. We hypothesize that as reported earlier for bone marrow cells (26), the absence of C38 methylation in tRNA^Asp (GTC)^ reduced accurate polypeptide synthesis, resulting in the observed phenotype in nonstressed parasites (Fig. 3C). In the context of this work, it is important to note that the glycolytic pathway is known to link metabolism with epigenetics by affecting the availability of cofactors required for enzymes involved in epigenetic modifications (39). For example, metabolic rewiring in cancer has profound effects on epigenetic gene regulation (40). This may explain how many proteins of the asexual blood stage that do not use GAC codons are up- or downregulated because of an initial downregulation of codon-biased glycolytic proteins in DNMT2KO parasites.

Bioinformatic analysis of GAC codon-containing genes transcribed during asexual blood-stage development predicted that the synthesis of many proteins (>400) may be affected in mutant parasites during the 48-h developmental cycle (Table S2). As a proof of principle, we investigated the expression of the Pf-Alba 4 gene, which is highly enriched for GAC codons (6 GAC codons), using a specific anti-Pf-Alba4 antibody (41). The Pf-Alba4 protein level was greatly reduced in mutant parasite extracts compared to that in the 3D7 wild type. Pf-Alba4 is an RNA-binding protein, and its deregulation may directly affect mRNA stability, thereby also contributing to changes in expression levels of protein pathways that do not depend on GAC codons.

Given that gene ontology analysis predicts a shift in metabolic pathways in Pf-DNMT2KO parasites, we investigated if mutant parasites are primed for being more sensitive to cellular stress situations. We compared 3D7 and Pf-DNMT2 mutant parasites grown under normal conditions and metabolically stressed conditions. We used a nutrient depletion method that is commonly used to induce sexual commitment in P. falciparum. This assay revealed a striking phenotype in Pf-DNMT2KO parasites linked to the transmission of this pathogen. We observed >6-fold induction of gametocytes. Further analysis revealed that the upregulation of the master regulator gene of sexual commitment, the ap2-g gene (>5-fold), is at the origin of this phenotype. ap2-g is silenced via Pf-HP1-mediated facultative heterochromatin and follows an intrinsic predetermined activation rate in a small subset of parasites at each onset of the asexual blood stage cycle (30; for a review, see reference 42). The switch rate can be modulated by environmental factors or inactivation of genes that interfere with heterochromatin formation at the ap2-g locus (30, 43) and is regulated by noncoding RNA originating at the GDV1 gene locus (5). Our demonstration of a link between Pf-DNMT2 and ap2-g activation is unprecedented and raises the issue of the association of cytosine methylation of tRNA^Asp (GTC)^ and perturbance of the homeostasis of sexual commitment. Heterochromatin-mediated silencing of ap2-g appears to be particularly sensitive to environmental changes (42, 44, 45). Since the interplay between metabolism and epigenetics is now well established, we hypothesize that a shift in the glycolytic pathway may prime Pf-DNMT2KO parasites for higher sexual commitment rates under nutrient stress. On the other hand, RNA-binding proteins that show Pf-DNMT2-dependent expression could also contribute to this phenotype. Given that multiple protein pathways are modulated at the same time by Pf-DNMT2, it is difficult to pin down a single mechanism causing increased sexual commitment at this stage of the analysis. However, our proteome analysis provides a novel set of data that will benefit the malaria community and, in particular, laboratories interested in the study of the complex cellular mechanisms that drive sexual commitment.

Another original phenotype emerged when we studied the fate of tRNA^Asp (GTC)^ in nutrient-depleted stressed versus nonstressed mutant parasites. Using a specific probe for tRNA^Asp (GTC)^, we showed that metabolic stress reduced dramatically the detectable levels of this tRNA (by 75%) in Northern blot analysis. The appearance of numerous smaller fragments in tRNA^Asp (GTC)^ from stressed parasites suggests that the C38 methylation protects this particular tRNA from an endonucleolytic degradation process that is activated under cellular stress. No changes of tRNA^Asp (GTC)^ levels were observed in the 3D7 wild type under normal or stressed conditions. tRNA cleavage under oxidative stress is a well-known process in eukaryotic model systems (46), and the role of DNMT2 in protecting tRNA from endonuclease-mediated cleavage has been shown in *Drosophila* and mouse models (32). A well-known endonuclease that mediates tRNA cleavage is angiogenin, with a particular role in cutting preferentially at the anticodon loop adjacent to the C38 position (47). No homologous angiogenin gene was found in the P. falciparum database PlasmoDB. Furthermore, it remains unknown if tRNA^Asp (GTC)^-derived fragments in Pf-DNMT2 mutants may have regulatory functions in gene expression as has been reported for a number of eukaryotes (48, 49). This observation opens a new field of research to investigate the tRNA^Asp (GTC)^ nuclease cleavage and the potential role in protein expression in this pathogen.

### Conclusion.

Here, we identified a specific writer of cytosine methylation of tRNA that safeguards homeostasis of malaria parasite protein synthesis. Pf-DNMT2KO parasites show a shift in metabolic pathways, such as glycolysis, that prime parasites for being destabilized by certain types of cellular stress identified in this work. The biology of reversible and dynamic tRNA modifications and their corresponding writers have been studied widely in several organisms (50). Exploring the tRNA epitranscriptome in the malaria parasite may reveal novel principles in response to host-induced stressor during the life cycle and reveal new targets for intervention strategies to combat this pathogen.

## MATERIALS AND METHODS

### Plasmid design.

To generate Pf-DNMT2 conditional knockout, plasmid pl6-gfp carrying the human dihydrofolate reductase (*hDHFR*) selectable marker was modified by replacing the enhanced green fluorescent protein (eGFP) box with a synthetic sequence including homology box 1, a first loxP site, the recodonized DNMT2 active domain, the artificial intron containing the second loxP site, and homology box 2. The synthetic recodonized sequence was ordered from GenScript and inserted into pl6-gfp using In-Fusion cloning between the restriction sites AflII (CTTAAG) and SpeI (ACTAGT). A DNMT2-specific guide RNA (TGTCTTGTATATGGCTGACA) was also cloned into modified pl6, yielding pl7-DNMT2-loxP plasmid.

To generate DNMT2 episomal expression, the Pf-DNMT2 open reading frame (ORF) was PCR amplified and cloned into the *pln-3HA* episome containing the blasticidin (BSD) selectable marker. Transfected parasites were maintained in culture in the presence of BSD at a final concentration of 5 μg/ml (for information on primers and sequences, see Text S1).

10.1128/mBio.02558-21.1 TEXT S1 Additional details on primers and sequences as well as some materials and methods used in the study. Download Text S1, DOCX file, 0.3 MB. Copyright © 2021 Hammam et al. 2021 Hammam et al. https://creativecommons.org/licenses/by/4.0/ This content is distributed under the terms of the Creative Commons Attribution 4.0 International license.

### Parasite culture and transfection.

P. falciparum 3D7A DiCre parasites were maintained in culture and synchronized as described previously (51). A total of 100 μg of pUF1-Cas9 (carrying the Cas9 expression cassette and the yeast *dihydrofolate dehydrogenase* selectable marker) and pl7-DNMT2-loxP were cotransfected in synchronous ring-stage parasites at 5% parasitemia. Transfected parasites were selected using 2.5 nM WR99210 and 1 μM DSM1. Transfectants were obtained after 40 days, and plasmid integration was verified by PCR. To induce the gene deletion, 100 nM rapamycin was added to synchronous ring-stage cultures for 4 h. The culture was then washed, and deletion was verified by PCR in the next cycle (for information on primers and sequences, see Text S1).

### Parasite growth analysis.

3D7-WT and the two DNMT2KO clones H9 and E11 were tightly synchronized as previously described (51). Parasites were then cultured at low parasitemia in blood from three different donors (three biological replicates/strain) for 4 days. Parasites were fixed on days 0 to 4 in phosphate-buffered saline (PBS)-glutaraldehyde (0.0025%) and quenched with 15 mM NH_4_CL-PBS. Parasitemia on each day was measured using flow cytometry (Guava; Merck) after staining of parasites with 0.5× SYBR green, and the results were plotted using GraphPad Prism 8.

### *In vitro* gametocyte induction and exflagellation assays.

Synchronous gametocyte induction in 3D7-WT and Pf-DNMT2 mutants was carried out as described previously (27), with minor modifications (see Text S1 for more details). Conversion rates were calculated by dividing percent gametocytemia on day 6 by percent parasitemia in rings on day 0 and multiplying the resulting value by 100.

### Asexual parasite proliferation in the presence of DHA.

3D7-WT and Pf-DNMT2 KO clones H9 and E11 were synchronized, and cultures containing 0.5% rings were incubated at 2% final hematocrit, with serial 7-point, 2-step dilutions of dihydroartemisinin (DHA) (starting concentration: 50 nM) for 72 h. Parasite proliferation was determined using SYBR green assay as described previously (52). DHA IC_50_ was calculated from three independent experiments using GraphPad Prism 8.

### qRT-PCR quantification of ap2-g gene expression.

RNA was harvested from synchronous parasites after lysis in 0.075% saponin in PBS and resuspension in QIAzol reagent. Total RNA was extracted using the miRNeasy minikit and performing on-column DNase treatment (Qiagen). Reverse transcription was achieved using SuperScript VILO (Thermo Fisher Scientific) and random hexamer primers. cDNA levels were quantified by quantitative PCR in the CFX384 real-time PCR detection system (Bio-Rad) using Power SYBR green PCR master mix (Applied Biosystems) and primers with sequences indicated below. Starting quantity means of ap2-g and the housekeeping reference gene UCE (ubiquitin-conjugating enzyme E2; *PF3D7_0812600*) of three or four independent biological replicates were extrapolated from a standard curve of serial dilutions of genomic DNA. Gene expression levels of ap2-g were normalized to UCE levels and expression fold change in DNMT2KO clones compared to 3D7-WT were plotted using GraphPad Prism 8. Primers were as follows: ap2-g forward primer, TCGAATGGGAAGAGAGCATGC; ap2-g reverse primer, CGCTTTCTTGTCCATGCAAC; UCE forward primer, TAACAGCCCAGCGAATCAAG; and UCE reverse primer, CGGCATCTTCTTCAGCTTTCTG.

### ELISA-based DNA 5mC quantification assay.

5mC levels in P. falciparum genomic DNA were quantified using the methylated DNA quantification kit from Abcam as described earlier (7) and following manufacturer’s instructions. 5mC percentage in each sample was calculated after plotting a standard curve in Microsoft Excel.

### Quantification of 5mdC in Plasmodium falciparum genomic DNA by LC/MS-MS.

Purified P. falciparum gDNA was denatured and digested using nuclease P1 (Sigma; N-8630). Analysis of global levels of 5mdC was performed on a Q Exactive mass spectrometer (Thermo Fisher Scientific) equipped with an electrospray ionization source (H-ESI II probe) coupled to an Ultimate 3000 RS high-performance liquid chromatograph (HPLC; Thermo Fisher Scientific). Digested DNA was injected onto a Thermo Fisher Hypersil Gold aQ chromatography column (see Text S1 for more details).

### tRNA extraction and hydrolysis.

To minimize host RNA contamination, parasites were purified by lysing human RBC with 0.15% saponin (Sigma) to selectively rupture RBC membranes, and the released parasites were washed three times with ice‐cold PBS. Purified parasites were homogenized with 5 volumes of TRIzol reagent (Invitrogen). Chloroform (Sigma) was added to the TRIzol‐homogenized cell lysates at one‐fifth lysate volume and the mixture was incubated at ambient temperature for 3 min. The mixture was centrifuged at 12,000 × *g* for 15 min at 4°C, and the aqueous phase was collected. Next, the aqueous phase was adjusted to 35% (vol/vol) ethanol, followed by RNA extraction using the PureLink microRNA (miRNA) isolation kit (Invitrogen) according to the manufacturer’s instructions. A sequential isolation protocol was adopted to enrich the yield of small RNA species using 70% (vol/vol) ethanol (Merck) for further experiments. The quantity and quality of RNA were assessed using a Bioanalyzer with RNA 6000 small RNA chips. Purified P. falciparum tRNA was hydrolyzed enzymatically as described previously (11), with a modified protocol using the following components in the buffer mix: 10 mM Tris-HCl (pH 7.9), 1 mM MgCl_2_, 5 U of Benzonase (Merck; no. 71206), 50 μM desferroxamine (Sigma; no. D9533), 0.1 μg μl^−1^ of pentostatin (Sigma; no. SML0508), 100 μM butylated hydroxytoluene (Sigma; no. W218405), 0.5 μg μl^−1^ of tetrahydrouridine (Calbiochem; no. 584222), 5 U of bacterial alkaline phosphatase (Thermo Fisher; no. 18011015), and 0.05 U of phosphodiesterase I (Sigma; no. P3243).

### LC/MS-MS analysis of tRNA modifications.

A Hypersil GOLD aQ column (100 by 2.1 mm, 1.9 μm [Thermo Scientific; no. 25305]) was used to resolve the digested ribonucleosides in a two-buffer eluent system (buffer A was 0.1% formic acid in water; buffer B was 0.1% formic acid in acetonitrile) (see Text S1 for more details).

### tRNA bisulfite sequencing library preparation and analysis.

Total RNA was extracted from synchronous (30 to 36 h postinfection [hpi]) parasites and the tRNA population was enriched as described earlier, using the PureLink miRNA isolation kit (Invitrogen) according to the manufacturer’s instructions. RNA bisulfite conversion was carried out using the Methylamp RNA bisulfite conversion kit (Epigentek). Briefly, RNA was mixed with the bisulfite conversion solution and samples were incubated in a thermal cycler using the following program: 65°C for 5 min, 60°C for 90 min, and 4°C hold. Bisulfite converted tRNA was cleaned up and eluted according to the manufacturer’s instructions. Treated RNA was further processed using the TruSeq RNA library prep kit (Illumina) according to the manufacturer’s instructions, with minor modifications (RNA was denatured at 94°C for 8 min and library amplification was performed using KAPA Hifi polymerase [KAPA Biosystems]). RNA BS libraries from 3D7-WT and DNMT2KO parasites were multiplexed and paired-end sequenced on an Illumina NextSeq 500.

m^5^C methylation sites were annotated using the meRanTk pipeline (53). Briefly, paired sequencing reads were aligned against the P. falciparum genome (version 43 [54]) using HiSat2 (55) implemented in meRanGh. Methylation sites were then called using meRanCall and filtered using bedtools “multiinter” and “intersect” (56) for those that were independently called in all replicates (*n* = 4 for 3D7-WT and *n* = 2 for each Pf-DNMT2KO clone). In addition, for the PF-DNMT2KO condition, only those sites called in both replicates of both clones were retained (Table S1). For heat map visualizations, the number of T conversions at each cytosine site of all reads aligning to PF3D7_0714700 was counted using the output of samtools “tview” (57). Heat maps were visualized in R using package ggplot2.

### Northern blot analysis of Asp (GTC) tRNA stability.

Total RNA was harvested from highly synchronous trophozoite stage 3D7-WT and DNMT2 mutant parasites (from both nonstressed and stressed conditions) and separated on 15% Tris-borate-EDTA (TBE) urea gels in order to better resolve the small RNA population. RNA was transferred to positively charged nylon membranes sandwiched by 0.5× TBE-soaked blotting papers, using Trans-Blot Turbo (Bio-Rad) (45 min at 300 mA). RNA membranes were then UV cross-linked and baked for 30 min at 80°C. RNA membranes were blocked and incubated with specific 3′-Asp (GTC) tRNA (CTCCGAGACCGGGAATTGAACCCGGGTCTTC-biotin) or 5S rRNA (CAGAGTTCTGATGGGATCTGGTGTGGCC-biotin) biotinylated DNA probes overnight at 42°C in ULTRAhyb ultrasensitive hybridization buffer (Ambion). Washes were carried out according to ULTRAhyb user guide instructions, and probes were detected using stabilized streptavidin-HRP conjugates following the chemiluminescent nucleic acid detection module (Thermo Scientific).

### Proteomics: protein extraction and digestion.

Parasite infected red blood cells were pelleted at 2,200 × *g* for 4 min at ambient temperature and subsequently lysed with ice-cold 0.15% (wt/vol) saponin (Sigma). The lysate was thoroughly mixed using an aspiration pipette and incubated on ice for 10 min to ensure complete lysis of the RBC membrane. The parasite pellet was centrifuged at 4,000 × *g* for 10 min at minimum deceleration, washed twice with ice-cold PBS, and frozen at −80°C. The parasite pellet was resuspended in a 6× volume of 8 M urea containing 1 mM sodium orthovanadate and homogenized using a sonicator pulse for 3 min at 25% amplitude and 2-s-on, 3-s-off pulse time. The lysate was spun at 16,000 × *g* at 4°C for 30 min to pellet the insoluble fraction, and the lysate was transferred into a new tube. Protein (100 μg) was reduced with 10 mM dithiothreitol (DTT) at 56°C for 1 h, followed by reduction using 100 mM iodoacetamide (IAA) for 1 h in the dark. This solution was diluted to 1 M urea and digested with 2 μg of trypsin (Thermo) overnight at ambient temperature. The resulting peptides were desalted using Pierce desalting columns as per the manufacturer’s instructions. These peptides were reconstituted in triethylammonium bicarbonate (TEAB) and labeled using tandem mass tag (TMT) labels (Thermo) as per the manufacturer’s instructions. The labeled peptides were combined, dried, and reconstituted in 0.1% formic acid (FA). After checking for labeling efficiency, the same peptides were then further fractionated using high-pH fractionation columns (Pierce) as per the manufacturer’s instructions into 8 fractions.

### LC/MS-MS and data analysis for proteomics.

Peptides were separated by reverse-phase HPLC (Thermo Easy nLC1000) using a precolumn (Thermo) and a self-pack 5-μm tip analytical column (15 cm by 5 μm C_18_; New Objective) over a 140 min gradient before nanoelectrospray using a Q Exactive HF-X mass spectrometer (Thermo). The mass spectrometer was operated in a data-dependent mode (see Text S1 for more details).

### Western blot analysis.

For the analysis of Pf-Alba4 expression, 3D7-WT and DNMT2 mutants were synchronized and total extracts were prepared from two biological replicates of each strain. For the analysis of DNMT2 episomal expression, parasites were synchronized and harvested at the ring, trophozoite, and schizont stages, and cytoplasmic and nuclear extracts were prepared as previously described (7). iRBC pellets (3 × 10^7^ parasites) were saponin lysed and total protein extracts were prepared by resuspending parasite pellets in extraction buffer (2× Laemmli buffer, protease inhibitor cocktail, 1 mM DTT). Lysates were sonicated for 10 min (HIGH 30 s ON/OFF), snap-frozen, and then stored at −80°C. Equal amounts of protein lysates were separated on Mini protean TGX stain-free precast gels from BIO-RAD and transferred to polyvinylidene difluoride (PVDF) membranes (Trans-Blot Turbo transfer pack; Bio-Rad). Membranes were blocked and then incubated with specific anti-Pf-Alba4 or anti-HA antibodies (12ca5 from Roche) overnight (ON) at 4°C. Pf-histone H3 (ab1791), Pf-aldolase (ab38905), and Pf-HSP70 (SPC-186) antibodies were used as controls. Next, membranes were washed and incubated with the corresponding secondary antibodies for 1 h at room temperature. Signal detection was carried out using Thermo Scientific SuperSignal West Pico chemiluminescent substrate.

### Immunofluorescence analysis of DNMT2 subcellular localization.

iRBC infected with parasites expressing the DNMT2-3HA episome were fixed with 0.0075% glutaraldehyde and 4% paraformaldehyde in PBS for 30 min at room temperature. Free aldehydes were quenched with 50 mM NH_4_Cl for 10 min at room temperature, and iRBC were permeabilized with 0.1% Triton X-100 for 15 min at room temperature. Permeabilized iRBC were then incubated with primary rat anti-HA antibody (Roche; 3F10) ON at 4°C. After several washes in PBS–0.05% Tween 20, goat anti-rat antibody conjugated to Alexa Fluor 647 was added for 45 min at room temperature. Samples were washed, 5 μl of cells was mounted with Vectashield fluorescence mounting medium, and images were acquired on a Deltavision Elite imaging microscope (GE Healthcare). Images were processed using ImageJ software.

### Statistics.

Statistical analysis was conducted in GraphPad Prism 8 using, unless otherwise specified, unpaired two-tailed *t* tests.

### Data availability.

All the proteomics data can be found in PRIDE (PXD024769) and Table S4. All the raw tRNA mass spectrometry-based data can be found in the following repository: https://chorusproject.org/anonymous/download/experiment/-9216109305439230312. tRNA bisulfite sequencing reads are available at the NCBI Sequence Read Archive (SRA) under BioProject number PRJNA723151.
