# Supplementary material for: Malaria Parasite Stress Tolerance Is Regulated by DNMT2-Mediated tRNA Cytosine Methylation
Source: mBio. 2021 Nov 2;12(6):e02558-21. doi: 10.1128/mBio.02558-21 (PMC8561396; doi:10.1128/mBio.02558-21)
Supplement: FIG S1 [file mbio.02558-21-sf001.pdf]

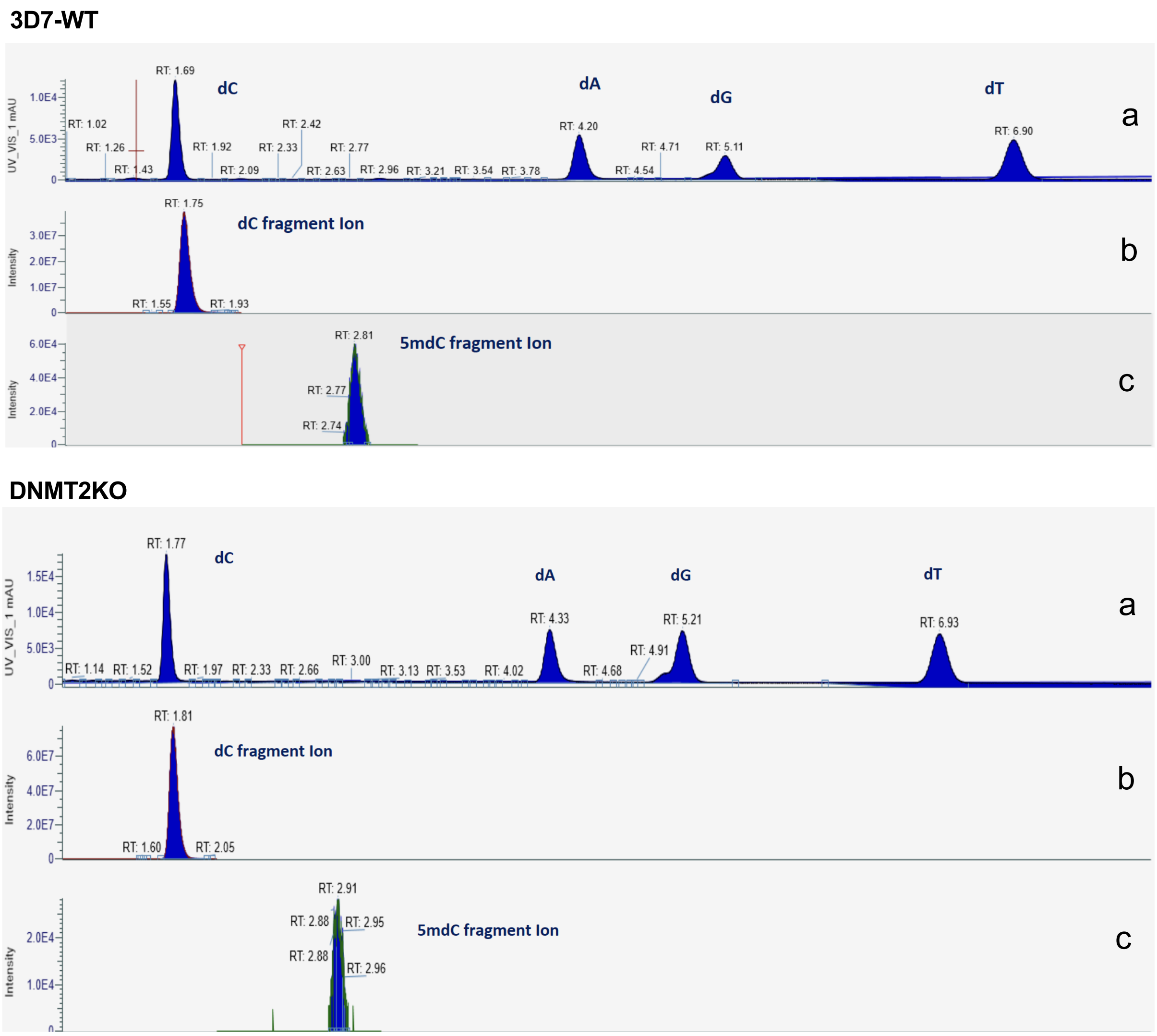

Figure S1: LC/MS-MS of *P. falciparum* WT and DNMT2KO genomic DNA for global methylation quantification.

a: Chromatogram of a typical injection of digested DNA from 3D7-WT (top) and DNMT2KO (bottom), showing deoxycytidine (dC), deoxyadenosine (dA), deoxyguanosine (dG) and thymidine (dT). Extracted fragment ion chromatogram, b: dC and c: 5-methyldeoxycytidine (5mdC). RT: retention time.
