# Supplementary material for: Malaria Parasite Stress Tolerance Is Regulated by DNMT2-Mediated tRNA Cytosine Methylation
Source: mBio. 2021 Nov 2;12(6):e02558-21. doi: 10.1128/mBio.02558-21 (PMC8561396; doi:10.1128/mBio.02558-21)
Supplement: FIG S2 [file mbio.02558-21-sf002.pdf]

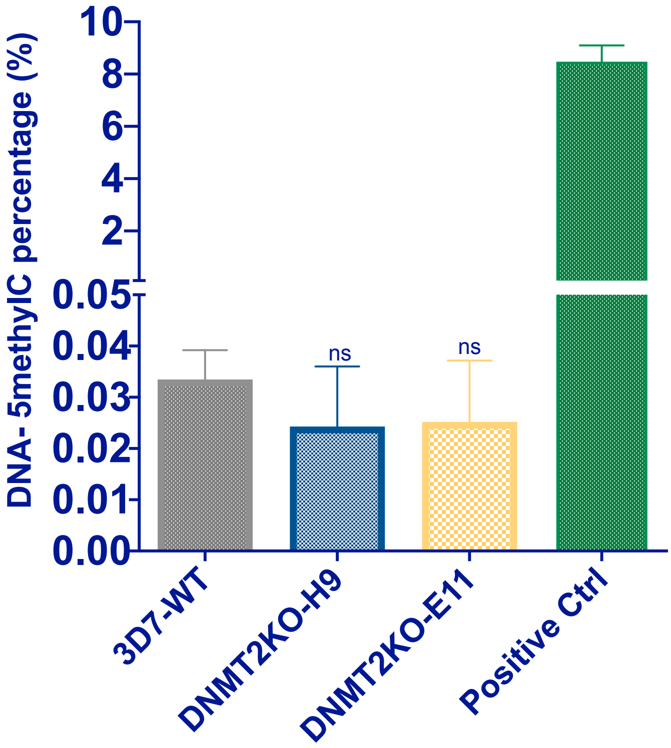

Figure S2: ELISA-based 5mC quantification in gDNA of 3D7-WT and the two DNMT2KO clones H9 and E11.

Parasites were synchronized at the schizont stage and gDNA was prepared from three independent biological replicates of each strain (3D7-WT, DNMT2KO-H9 and DNMT2KO-E11). Methylated DNA oligos were used as positive controls.
