# Supplementary material for: Malaria Parasite Stress Tolerance Is Regulated by DNMT2-Mediated tRNA Cytosine Methylation
Source: mBio. 2021 Nov 2;12(6):e02558-21. doi: 10.1128/mBio.02558-21 (PMC8561396; doi:10.1128/mBio.02558-21)
Supplement: FIG S3 [file mbio.02558-21-sf003.pdf]

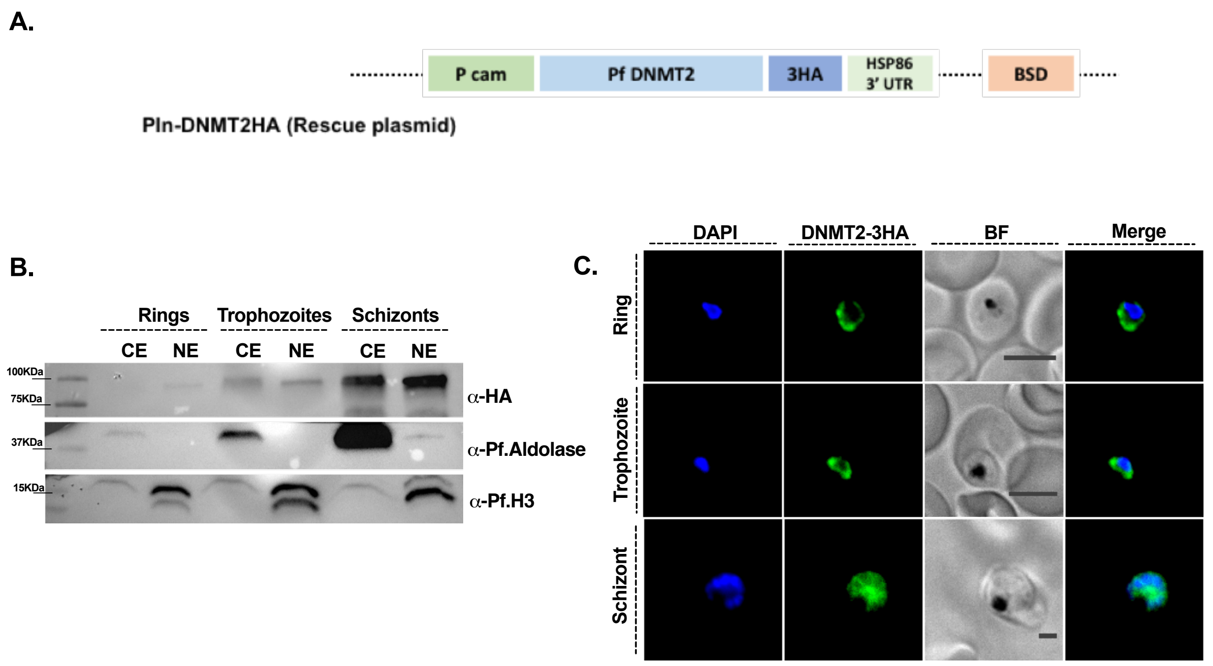

Figure S3: Episomal expression of Pf-DNMT2 reveals its dual cytoplasmic and nuclear subcellular localization in *P. falciparum*

(A) Schematic of pln-DNMT2-3HA episome. P cam: Pf-Calmodulin promoter; 3HA: 3xHA tag sequences; UTR: untranslated region; BSD: blasticidin selectable marker.

(B) Western blot analysis of Pf-DNMT2 expression in the cytoplasmic and nuclear fractions of synchronous rings, trophozoite and schizont stages parasites. Anti-HA antibodies were used to detect Pf-DNMT2 expression (expected size at 82KDa). Anti-Pf-histone H3 and Pf-aldolase antibodies were used as nuclear and cytoplasmic controls, respectively. CE: Cytoplasmic extracts; NE: nuclear extracts.

(C) Immunofluorescence assays using anti-HA antibodies. DAPI: nuclear markers; BF: bright field. Scale bar= 5um.
