# Supplementary material for: Malaria Parasite Stress Tolerance Is Regulated by DNMT2-Mediated tRNA Cytosine Methylation
Source: mBio. 2021 Nov 2;12(6):e02558-21. doi: 10.1128/mBio.02558-21 (PMC8561396; doi:10.1128/mBio.02558-21)
Supplement: FIG S4 [file mbio.02558-21-sf004.pdf]

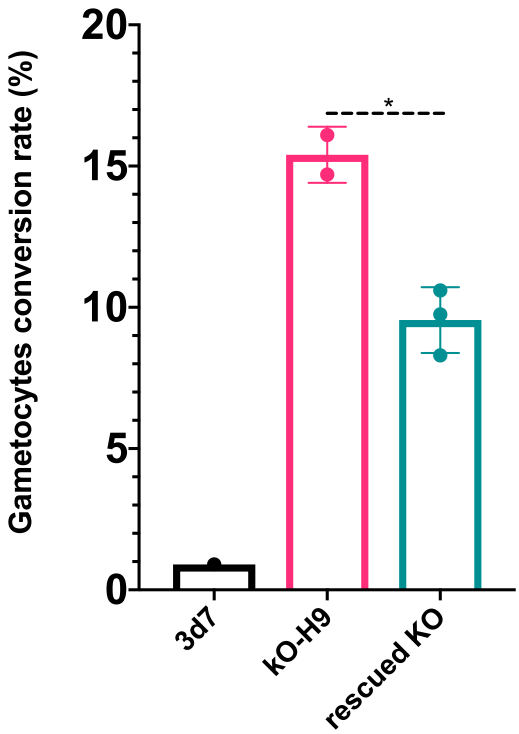

Figure S4: Episomal expression of Pf-DNMT2 partially restores the gametocyte conversion rate in the DNMT2 KO strain.

Full length DNMT2 was expressed in one of the two DNMT2KO clones (rescued KO) and gametocyte induction was carried out *in vitro* in the 3D7, KO-clone H9 and the rescued KO, as described earlier. Gametocyte conversion rate is calculated by dividing the gametocytemia (calculated using FACS double staining with SYBR green and Mitotracker) on day 6 post induction by the parasitemia in rings on day 0 pre-induction * 100. Statistical analysis was conducted using unpaired two tailed T-tests. *=p<0,05.
