# Supplementary material for: Malaria Parasite Stress Tolerance Is Regulated by DNMT2-Mediated tRNA Cytosine Methylation
Source: mBio. 2021 Nov 2;12(6):e02558-21. doi: 10.1128/mBio.02558-21 (PMC8561396; doi:10.1128/mBio.02558-21)
Supplement: FIG S5 [file mbio.02558-21-sf005.pdf]

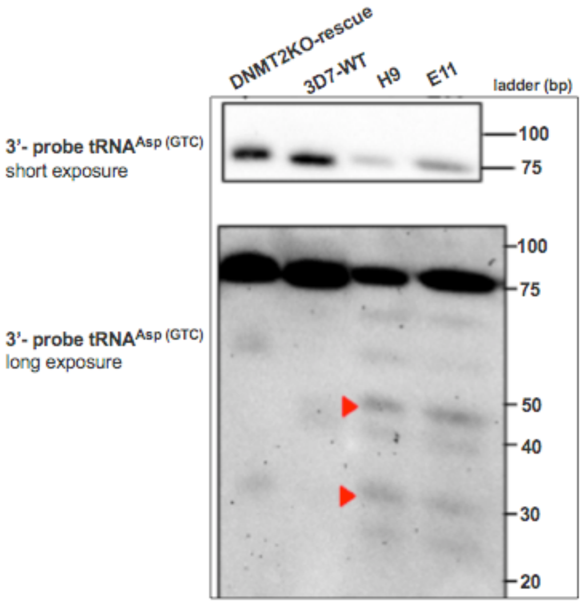

Figure S5: tRNA fragments are detectable in stressed DNMT2 mutants

Northern blot analysis of FL- tRNA$^{Asp\ (GTC)}$ abundance (top panel) and tRNA fragments (bottom panel) in DNMT2 mutants, 3D7-WT and rescued DNMT2-KO. Rescued DNMT2KO: DNMT2 KO clone H9 transfected with the episome expressing DNMT2; 3D7-WT: 3D7 wild type; E11 and H9 are the two mutant clones. Red arrows indicate tRNA fragments at around 30 and 45 bp. RNA ladder: DynaMarker® Prestain Marker for Small RNA Plus.
