# Supplementary material for: Malaria Parasite Stress Tolerance Is Regulated by DNMT2-Mediated tRNA Cytosine Methylation
Source: mBio. 2021 Nov 2;12(6):e02558-21. doi: 10.1128/mBio.02558-21 (PMC8561396; doi:10.1128/mBio.02558-21)
Supplement: FIG S6 [file mbio.02558-21-sf006.pdf]

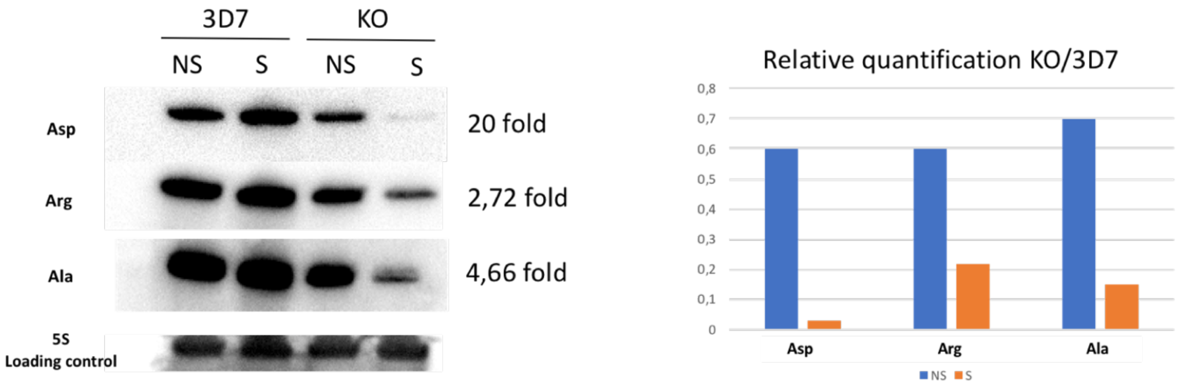

Figure S6: northern blot analysis of the abundance of tRNAs in DNMT2 mutants vs 3D7 WT.

The left panel shows the northern blots for the expression of aspartic acid (asp), arginine (arg) and alanine (ala) tRNAs in 3D7-wild type and the DNMT2KO parasites, in both non-stressed(NS) and stressed (S) conditions. 5S rRNA was used as RNA loading control. The right panel shows the relative quantification of the corresponding tRNA bands.
