# Supplementary material for: Malaria Parasite Stress Tolerance Is Regulated by DNMT2-Mediated tRNA Cytosine Methylation
Source: mBio. 2021 Nov 2;12(6):e02558-21. doi: 10.1128/mBio.02558-21 (PMC8561396; doi:10.1128/mBio.02558-21)
Supplement: TEXT S1 [file mbio.02558-21-s0001.docx]

**Malaria parasites stress tolerance is regulated by DNMT2 mediated tRNA cytosine methylation**

**Supplementary materials**

**Primers and sequences**

**
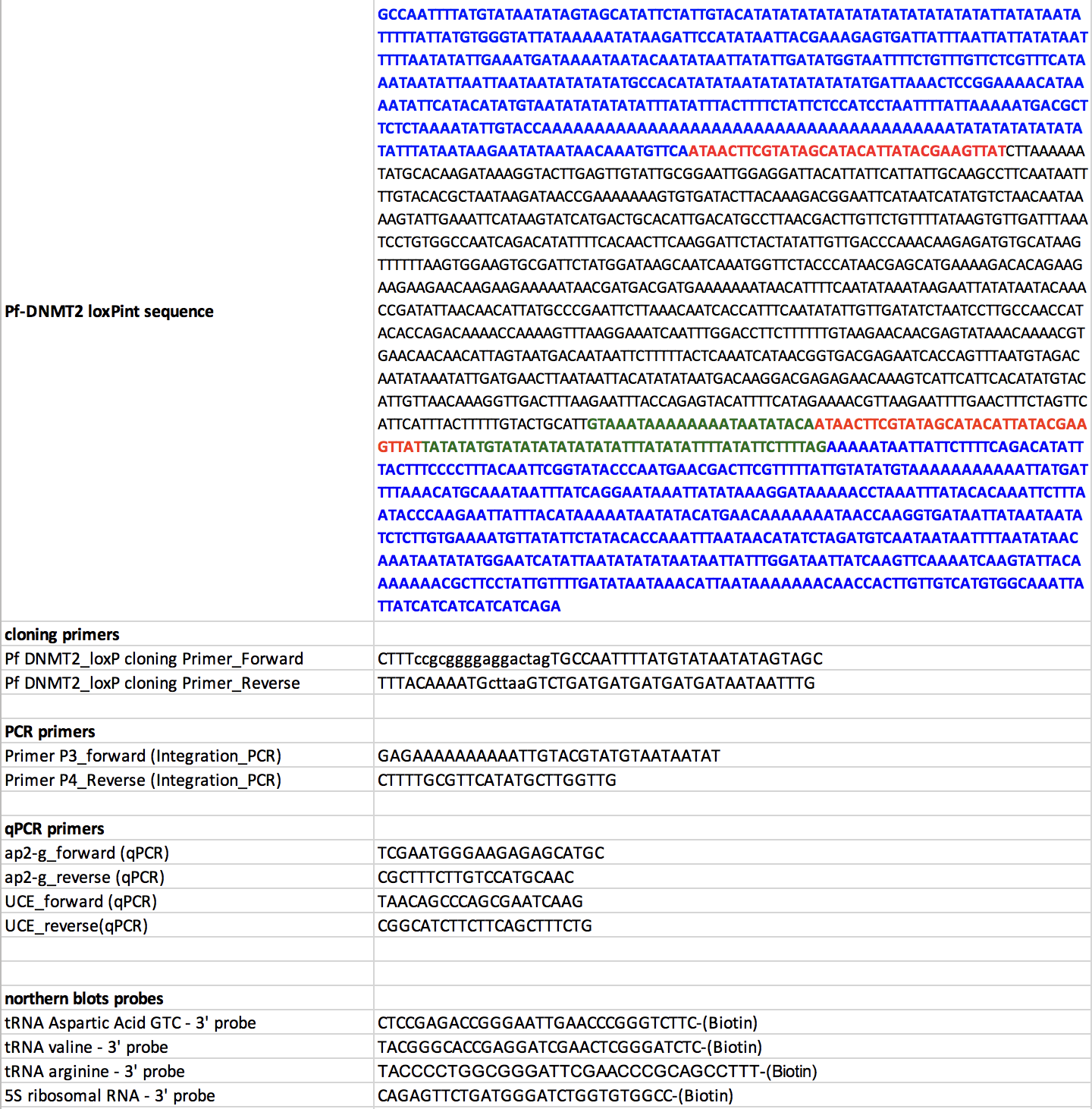
**

**In vitro Gametocyte induction and exflagellation assays**

Trophozoite purification steps performed using MACS columns in (^17^) were done using gelatine flotation (Plasmion®). Also, media containing 0.05% Albumax I was used until day 0 of induction (corresponding to gametocyte-committed ring stage parasites) then followed by the use of media containing 0.25% (v/v) human serum and 0.025% (w/v) Albumax I. N-acetylglucosamine was added for 5 days to get rid of asexual parasites population. A small fraction of the culture was used to estimate gametocytemia on day 6 post-induction (gametocytes stage III with no asexual parasites left) using flow cytometry (Guava, Merck) with a method adapted from (^18^). Briefly, gametocytes were stained with 0.5X SYBR-Green I (DNA, Invitrogen™ S7563) and 100nM Mitotracker Deep Red FM (Active mitochondria, Invitrogen™ M22426), the double positive population indicating viable gametocytes. This technique allowed us to accurately measure gametocytemia without use of reporter genes so that we can easily compare the induction rates in 3D7-WT and KO parasites. Gametocytemia was also verified by counting Giemsa-stained thin blood smears. Conversion rates were calculated by dividing gametocytemia (%) on day 6 by the parasitemia (%) in rings on day 0 *100. Gametocytes were kept in culture with daily changing of media until they reached maturity on day 11. In vitro exflagellation was carried out by mixing iRBC containing mature gametocytes, with human serum at 50% final haematocrit. 10 µl of this mix were deposited between slides and coverslips and the number of exflagellation centres per microscopic field (Objective 50X, Olympus) was counted in at least 10 different fields over the course of 30 minutes. Exflagellation relative to gametocytemia was obtained by dividing the mean number of exflagellation centers by the corresponding gametocytemia (%).

**Quantification of 5mdC in *Plasmodium falciparum* genomic DNA by LC/MS-MS.**

Purified *P. falciparum* genomic DNA sample was denatured by boiling for 3 min. The sample was cooled rapidly on ice and digestion was done in a mix containing 0.1 M ammonium acetate (pH5.3), 10 mM ZnCl_2_, 50 µM deferoxamine (Sigma D9533), 100 µM butylated hydroxytoluene (Sigma Chemical Co W218405), 0.5 µg/µL tetrahydrouridine (Calbiochem 584222). After incubating with 2 U nuclease P1 (Sigma N-8630) at 45°C for 2h, 3 mUnits phosphodiesterase I (Sigma Aldrich P3243-1VL) was added and incubated at 37°C for a further 2h. After incubating with 2 U Calf Intestinal Alkaline Phosphatase (Biolabs M0290) at 37°C for 2 h, reaction was quenched by adding 20 µL sodium acetate 30 mM. Sample are then filtered in a microspin filter before LC/MS-MS analysis. Nucleoside analysis was performed on a Q exactive mass spectrometer (Thermo Fisher Scientific), equipped with an electrospray ionization source (H-ESI II Probe) coupled with an Ultimate 3000 RS HPLC (Thermo Fisher Scientific). Digested DNA was injected onto a Thermo Fisher Hypersil Gold aQ chromatography column (100 mm x 2.1 mm, 1.9 µm particle size) heated at 30°C. The flow rate was set at 0.3 mL/min and run with an isocratic eluent of 1% acetonitrile in water with 0.1 % formic acid during 10 min. Parent ions were fragmented in positive ion mode with 10 % normalized collision energy in parallel-reaction monitoring (PRM) mode. MS2 resolution was 17,500 with an AGC target of 2^e^5, a maximum injection time of 50 ms and an isolation window of 1.0 m/z. The inclusion list contained the following masses: dC (228.1), 5-mdC (242.1). Extracted ion chromatograms of base fragments (±5ppm) were used for detection and quantification (112.0506 Da for dC; 126.0662 Da for 5-mdC). Calibration curves were previously generated using synthetic standards in the ranges of 0.2 to 50 pmoles injected for dC and 0.02 to 10 pmoles for 5mdC. Results are expressed as a % of total dC*.*

**Preparation of nuclear extracts**

First, parasites pellet (10^9 parasites) is resuspended in 1ml of cytoplasmic lysis buffer (25mM Tris-HCl pH 7.5, 10mM NaCl, 1% Igepal, 1mM DTT, 1.5mM MgCl_2_ and protease inhibitor cocktail) and incubated at 4°C on rotation. Nuclei are sedimented by centrifugation at 16,000g for 20mins at 4°C, and supernatant containing the cytoplasmic fraction is aliquoted and snap frozen. For nuclear extraction, nuclei are resuspended in 100μl of nuclear lysis buffer (25mM Tris-HCl pH 7.5, 600 mM NaCl, 1% Igepal, 1mM DTT, 1.5mM MgCl_2_ and protease inhibitor cocktail) and shacked vigorously for 30 min at 4°C. Finally, 300μl of cytoplasmic lysis buffer are added and cell debris are pelleted by centrifugation at 20,000g for 20 mins at 4°C and supernatant containing the nuclear soluble proteins is aliquoted and snap frozen. Protein concentration is measured using Bradford colorimetric protein quantification assay.

**LC-MS/MS analysis of tRNA modifications**

HPLC was performed at a flow rate of 300 μl m−1 at 25 °C. The gradient of 0.1% formic acid in acetonitrile was as follows: 0–12 min, held at 0%; 12–15.3 min, 0–1%; 15.3–18.7 min, 1–6%; 18.7–20 min, held at 6%; 20–24 min, 6–100%; 24–27.3 min, held at 100%; 27.3–28 min, 100–0%; 28–41 min, 0%. The HPLC column was directly connected to an Agilent 6490 triple quadrupole mass spectrometer with ESI Jetstream ionization operated in positive ion mode. The voltages and source gas parameters were as follows: gas temperature, 50 °C; gas flow, 11 l min−1; nebulizer, 20 psi; sheath gas temperature, 300 °C; sheath gas flow, 12 l min−1; capillary voltage, 1,800 V; and nozzle voltage, 2,000 V. The molecular transition ions were quantified in multiple-reaction monitoring (MRM) mode using the parameters as described previously ^12^. LC-MS/MS data was extracted using the MassHunter Qualitative and Quantitative Analysis Software (version B06.00). To account for any background signal that could be a contribution from the salts and enzymes in the digestion buffer, the signal intensity for each ribonucleoside was subtracted from a matrix sample (digestion mix without tRNA) and normalized to the canonical nucleosides (rA, rU, rG, and rC).

**LC-MS/MS and data analysis for proteomics**

The parameters for the full scan MS were: resolution of 70,000 across 350-2000 m/z, AGC 3e6, and maximum IT 300 ms. The full MS scan was followed by MS/MS for the top 10 precursor ions in each cycle with a NCE of 28 (34 for TMT samples) and dynamic exclusion of 30 s. Raw mass spectral data files (.raw) were searched using Proteome Discoverer (Thermo). Sequest (REF) search parameters were: 10 ppm mass tolerance for precursor ions; 0.08Da for fragment ion mass tolerance; 2 missed cleavages of trypsin; fixed modification was carbamidomethylation of cysteine; variable modifications were lysine-labelled TMT residues, peptide N-terminal TMT labels, methionine oxidation and serine, threonine and tyrosine phosphorylation. Only peptides with a SEQUEST Xcorr score greater than or equal to 2 and an isolation interference less than or equal to 30 were included in the data analysis. For TMT samples, a minimum abundance of 500 was used a threshold in order to ensure robustness of data. PSM data was further processed using MSStatsTMT ^27^ in order to carry out a differential analysis. For interpretations of the relationships between codon usage (codon frequency) and up-regulated proteins at different time points, PLS-R was performed using NIPALS algorithm. The values of codon usage in synonymous codon choices of those proteins were retrieved from the pre-calculated genome-wide codon usage. Outliers which could cause over-fitting were removed by manual inspection of residual sample variances and leverages, as well as Hotelling’s T2 statistics. Marten’s uncertainty test with optimal number of PCs was used to cross-validate the PLS model. Eigenvector based multivariate statistics was performed using UnscramblerX (v10.3, Camo).
